# Supplementary material for: Microtubule Minus-End Binding Protein CAMSAP2 and Kinesin-14 Motor KIFC3 Control Dendritic Microtubule Organization
Source: Curr Biol. 2020 Mar 9;30(5):899–908.e6. doi: 10.1016/j.cub.2019.12.056 (PMC7063570; doi:10.1016/j.cub.2019.12.056)
Supplement: Document S1. Figures S1–S3 and Tables S1 and S2 [file mmc1.pdf]

Current Biology, Volume *30*

Supplemental Information

Microtubule Minus-End Binding Protein CAMSAP2 and Kinesin-14 Motor KIFC3 Control Dendritic Microtubule Organization

Yujie Cao, Joanna Lipka, Riccardo Stucchi, Mithila Burute, Xingxiu Pan, Sybren Portegies, Roderick Tas, Jelmer Willems, Lena Will, Harold MacGillavry, Maarten Altelaar, Lukas C. Kapitein, Martin Harterink, and Casper C. Hoogenraad

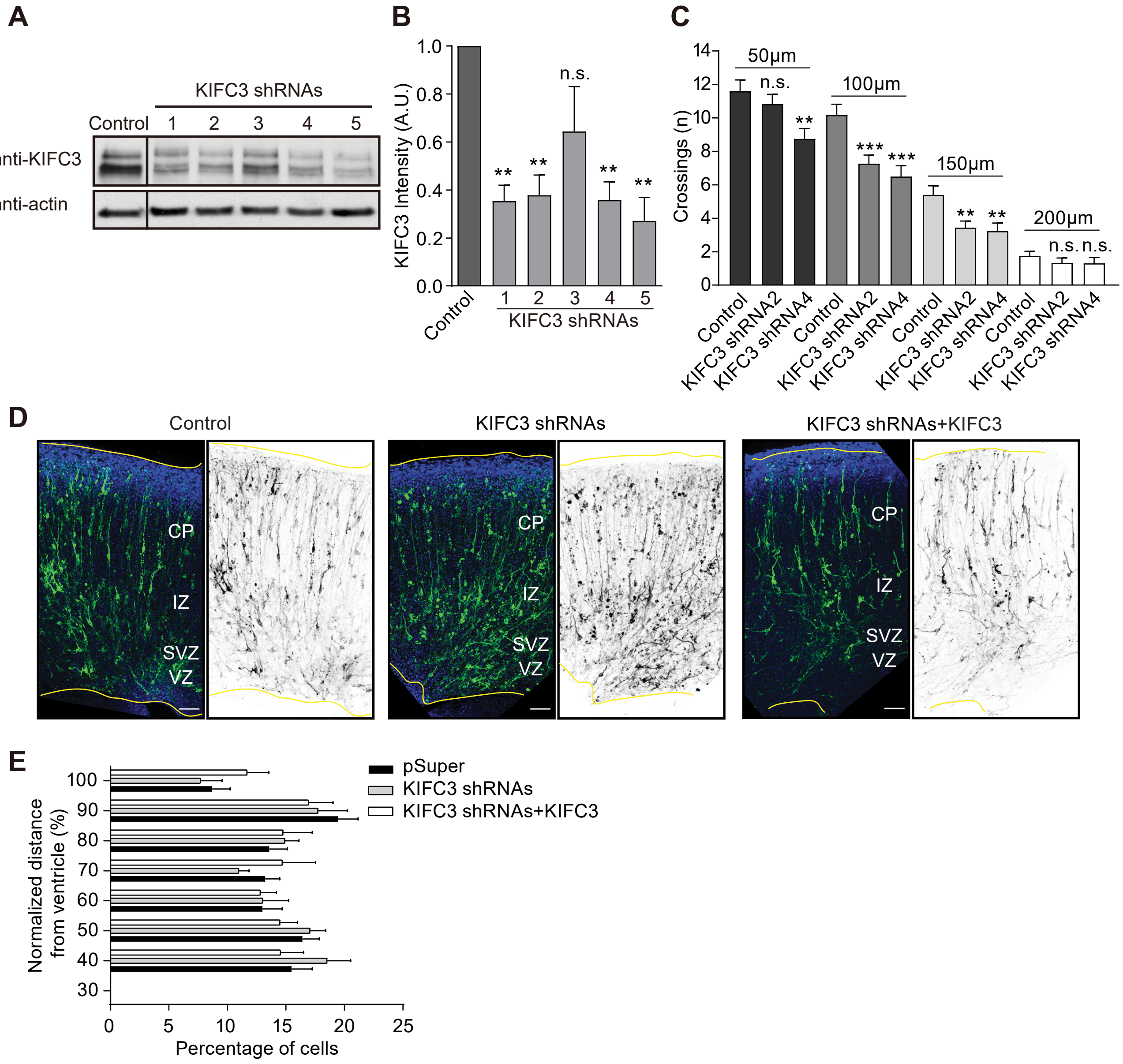

Figure S1. **KIFC3 is important for dendrite branching. Related to Figure 1.**

(A) Example of a western blot of protein extracts of DIV 7 hippocampal neurons electroporated at DIV 0 with pSuper-scrambled as Control or KIFC3-shRNA1, 2, 3, 4 and 5. Levels of actin were used as loading Control.

(B) Quantification of protein levels of KIFC3 of samples represented in (A) (N=3). Error bars represent SEM. $*p < 0.05$; $**p < 0.01$; $***\ p < 0.001$ (Unpared T-test).

(C) Quantification of crossings from the sholl analysis in Figure 1B at 50µm, 100µm, 150µm and 200µm positions. Error bars represent SEM. Unpared T-test was performed and columns were compared with corresponding Control. $*p < 0.05$; $**p < 0.01$; $***\ p < 0.001$.

(D-E) Representative images of mouse cortex after 4 days of *ex vivo* electroporated with GFP and pSuper-scrambled, KIFC3 shRNAs or KIFC3 shRNAs with KIFC3 as rescue. Left panels represent GFP and DAPI channels. DAPI is in blue and GFP is in Green. Pail surface at the top and ventricle at the bottom are outlined in yellow. CP, cortical plate; IZ, intermediate zone;SVZ, subventricular zone; VZ, ventricular zone. Right panels represent GFP only channel and GFP is in gray. Scale bars=50µm. Error bars represent SEM.

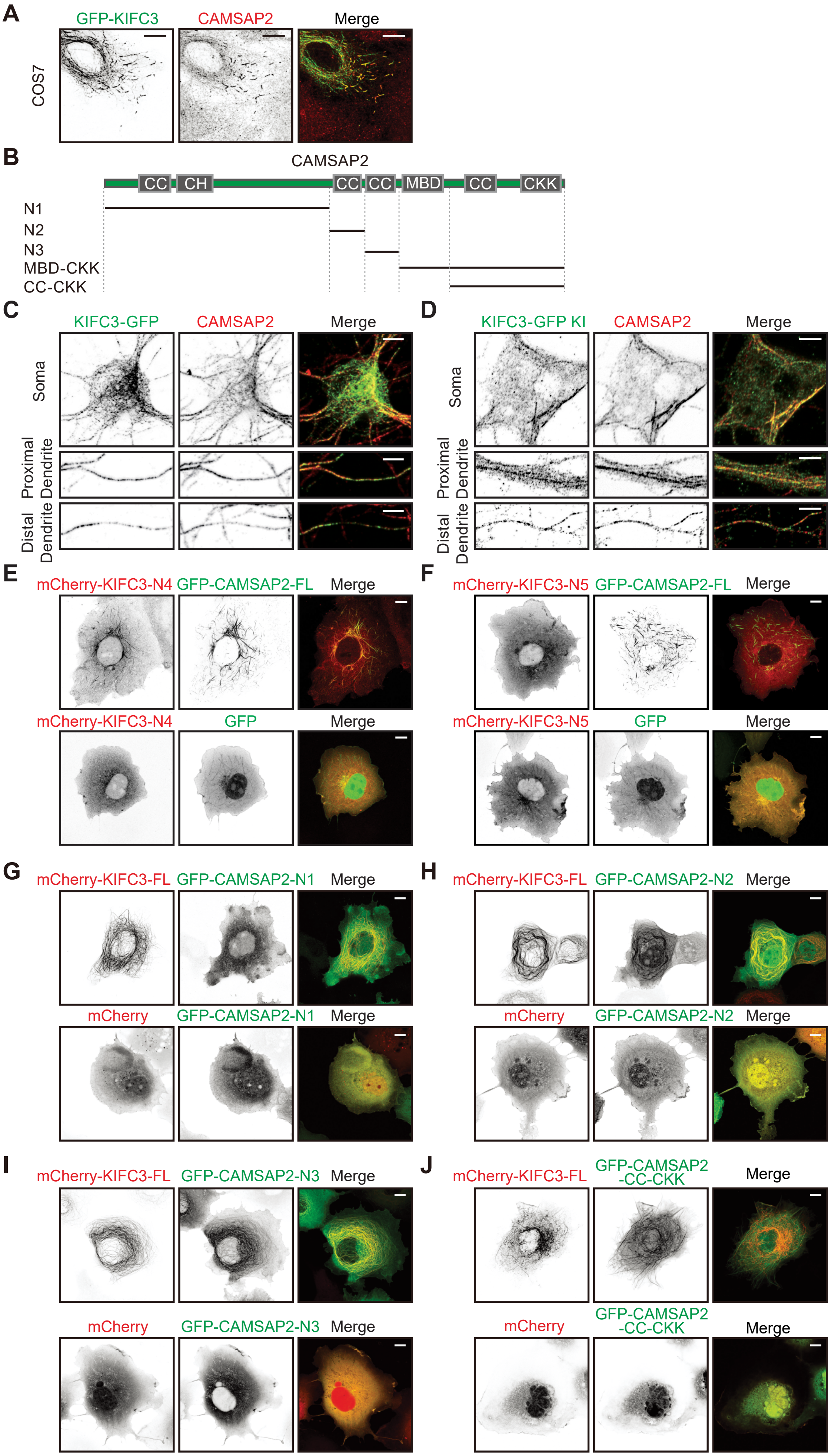
A
GFP-KIFC3
CAMSAP2
Merge
COS7
B
CAMSAP2
CC
CH
CC
CC
MBD
CC
CKK
N1
N2
N3
MBD-CKK
CC-CKK
C
KIFC3-GFP
CAMSAP2
Merge
Soma
Proximal Dendrite
Distal Dendrite
D
KIFC3-GFP KI
CAMSAP2
Merge
Soma
Proximal Dendrite
Distal Dendrite
E
mCherry-KIFC3-N4
GFP-CAMSAP2-FL
Merge
mCherry-KIFC3-N4
GFP
Merge
F
mCherry-KIFC3-N5
GFP-CAMSAP2-FL
Merge
mCherry-KIFC3-N5
GFP
Merge
G
mCherry-KIFC3-FL
GFP-CAMSAP2-N1
Merge
mCherry
GFP-CAMSAP2-N1
Merge
H
mCherry-KIFC3-FL
GFP-CAMSAP2-N2
Merge
mCherry
GFP-CAMSAP2-N2
Merge
I
mCherry-KIFC3-FL
GFP-CAMSAP2-N3
Merge
mCherry
GFP-CAMSAP2-N3
Merge
J
mCherry-KIFC3-FL
GFP-CAMSAP2 -CC-CKK
Merge
mCherry
GFP-CAMSAP2 -CC-CKK
Merge

Figure S2. KIFC3 interacts with CAMSAP2. Related to Figure 2.

(A) Representative images of a COS7 cell transfected with KIFC3-GFP and stained with CAMSAP2 antibody. Scale bar=10μm.

(B) Schematic representation of CAMSAP2 truncation constructs used. CC: coiled-coiled domain, MD: motor domain, CH: Calponin-homology domain, MBD: microtubule binding domain, CKK: CKK domain.

(C) Representative images of a DIV7 neuron transfected with KIFC3-GFP and stained with CAMSAP2 antibody. Scale bar=5μm.

(D) Representative images of a DIV11 neuron transfected with the HITI CRISPR GFP knock-in construct and probed for CAMSAP2 and GFP (to enhance the signal in transfected cells). Scale bar=5μm.

(E-F) Representative images of COS7 cells transfected with GFP-CAMSAP2-FL or GFP and co-transfected with mCherry-KIFC3-N4 or mCherry-KIFC3-N5. Scale bar=10μm.

(G-J) Representative images of COS7 cells transfected with either mCherry or mCherry-KIFC3 together with GFP tagged CAMSAP2 truncations. Scale bar=10μm.

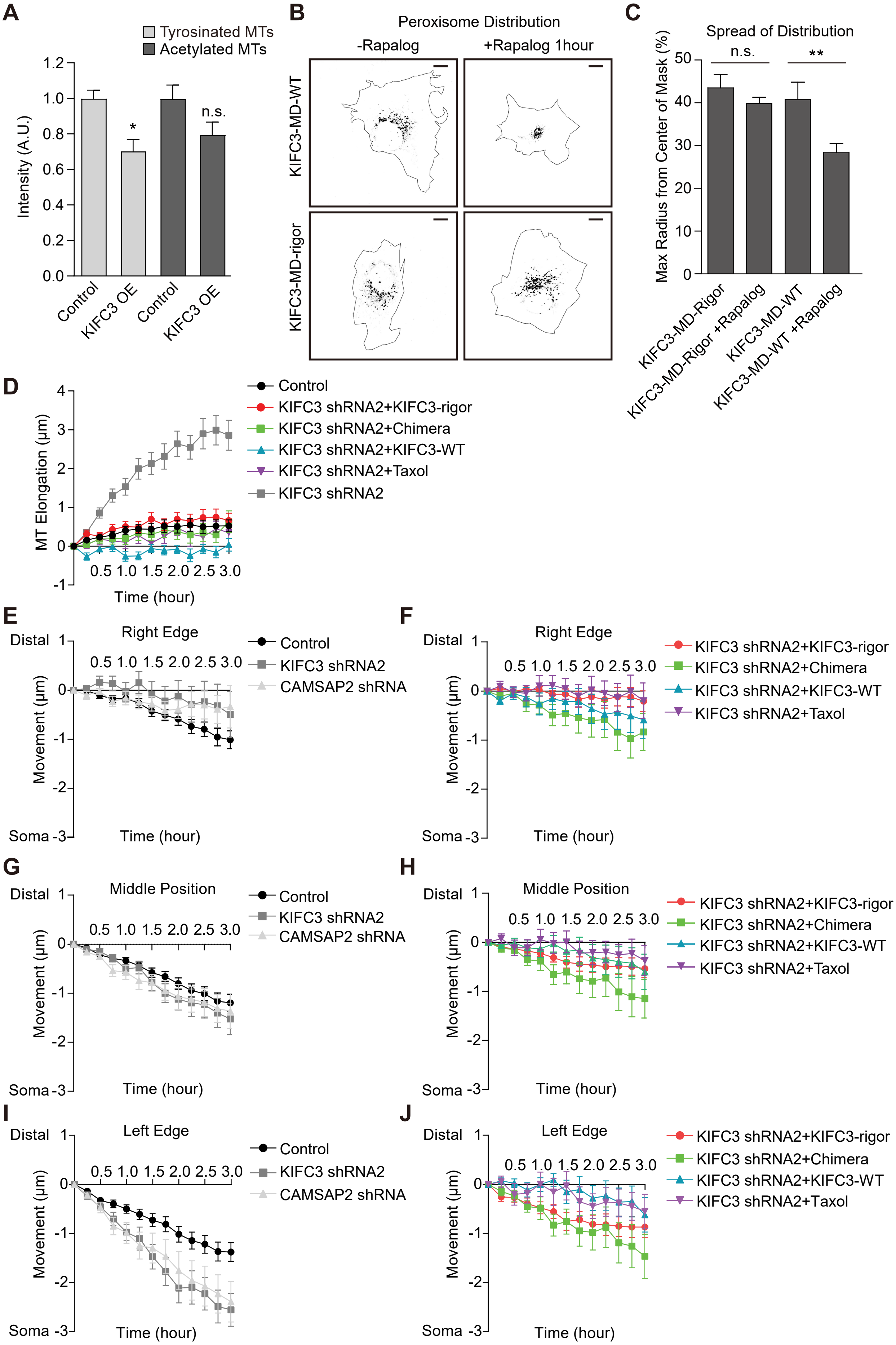
A
Tyrosinated MTs
Acetylated MTs
Intensity (A.U.)
1.2
1.0
0.8
0.6
0.4
0.2
0.0
*
n.s.
Control
KIFC3 OE
Control
KIFC3 OE
B
Peroxisome Distribution
-Rapalog
+Rapalog 1hour
KIFC3-MD-WT
KIFC3-MD-rigor
C
Spread of Distribution
Max Radius from Center of Mask (%)
50
40
30
20
10
0
n.s.
**
KIFC3-MD-Rigor
KIFC3-MD-Rigor +Rapalog
KIFC3-MD-WT
KIFC3-MD-WT +Rapalog
D
MT Elongation (μm)
4
3
2
1
0
-1
0.5 1.0 1.5 2.0 2.5 3.0
Time (hour)
Control
KIFC3 shRNA2+KIFC3-rigor
KIFC3 shRNA2+Chimera
KIFC3 shRNA2+KIFC3-WT
KIFC3 shRNA2+Taxol
KIFC3 shRNA2
E
Right Edge
Distal 1
0
-1
-2
Soma -3
Movement (μm)
0.5 1.0 1.5 2.0 2.5 3.0
Time (hour)
Control
KIFC3 shRNA2
CAMSAP2 shRNA
F
Right Edge
Distal 1
0
-1
-2
Soma -3
Movement (μm)
0.5 1.0 1.5 2.0 2.5 3.0
Time (hour)
KIFC3 shRNA2+KIFC3-rigor
KIFC3 shRNA2+Chimera
KIFC3 shRNA2+KIFC3-WT
KIFC3 shRNA2+Taxol
G
Middle Position
Distal 1
0
-1
-2
Soma -3
Movement (μm)
0.5 1.0 1.5 2.0 2.5 3.0
Time (hour)
Control
KIFC3 shRNA2
CAMSAP2 shRNA
H
Middle Position
Distal 1
0
-1
-2
Soma -3
Movement (μm)
0.5 1.0 1.5 2.0 2.5 3.0
Time (hour)
KIFC3 shRNA2+KIFC3-rigor
KIFC3 shRNA2+Chimera
KIFC3 shRNA2+KIFC3-WT
KIFC3 shRNA2+Taxol
I
Left Edge
Distal 1
0
-1
-2
Soma -3
Movement (μm)
0.5 1.0 1.5 2.0 2.5 3.0
Time (hour)
Control
KIFC3 shRNA2
CAMSAP2 shRNA
J
Left Edge
Distal 1
0
-1
-2
Soma -3
Movement (μm)
0.5 1.0 1.5 2.0 2.5 3.0
Time (hour)
KIFC3 shRNA2+KIFC3-rigor
KIFC3 shRNA2+Chimera
KIFC3 shRNA2+KIFC3-WT
KIFC3 shRNA2+Taxol

Figure S3. KIFC3-N1 shows dominant negative effect, KIFC3-rigor mutation validation and microtubule mobility rescue and quantification. Related to Figure 3 and Figure 4.

(A) Quantification of acetylated tubulin, tyrosinated tubulin levels in DIV11 neurons transfected with mCherry-KIFC3. Neighboring non-transfected neurons were used as Control. N=2. Control: n=22; mCherry-KIFC3: n=20. Error bars represent SEM. Unpared-T-test was performed and columns were compared with corresponding Control. $*p < 0.05$; $**p < 0.01$; $*** p < 0.001$.

(B) Representative images of COS7 cells transfected with either PEX-RFP-FKBP and KIFC3-CC-MD-GFP-FRB or PEX-RFP-FKBP and KIFC3-CC-MD Rigor (396T-N)-GFP-FRB. PEX-RFP-FKBP was used to visualize peroxisomes in cells. Scale bar=10 μm.

(C) Quantification of peroxisome redistribution in cells described in (B). KIFC3-MD-Rigor: N=1, n=7; KIFC3-MD-Rigor+Rapalog: N=1, n=6, KIFC3-MD-WT: N=1, n=6; KIFC3-MD-WT+Rapalog: N=1, n=12. Error bars represent SEM. $*p < 0.05$; $**p < 0.01$; $*** p < 0.001$ (Unpaired T-test).

(D) Quantification of microtubule elongation corresponding to Figure 4A, 4C and 4D. Control: N=5, n=36; KIFC3 shRNA2: N=4, n=35; KIFC3 shRNA2+KIFC3-rigor: N=2, n=20; KIFC3 shRNA2+KIFC3-Chimera: N=3, n=10; KIFC3 shRNA2+KIFC3-WT: N=3, n=12; KIFC3 shRNA2+Taxol: N=3, n=8. Error bars represent SEM.

(E-J) Quantification of microtubule movement of the left edge, right edge and middle position of the photo-converted region corresponding to Figure 4A, 4C and 4D. Error bars represent SEM.

		Control		KIFC3-FL		KIFC3-N1		KIFC3-N3		
**PROTID**	**Name**	**Peptides**	**PSM**	**Peptides**	**PSM**	**Peptides**	**PSM**	**Peptides**	**PSM**	
Q4KLH6	CEP162	0	0	9	17	10	21	9	17	MT/Centrosome
Q63560	Map6	0	0	9	12	0	0	0	0	MT
D4AEC2	Camsap2	0	0	7	7	4	4	0	0	MT
Q63170	Dnah7	0	0	17	27	14	20	0	0	Dynein-ralated
E9PSL8	Kif6	0	0	6	37	1	32	5	48	Kinesin
G3V6L4	Kif5c	1	1	4	5	4	4	4	5	Kinesin
D3Z9C7	Piccolo	0	0	14	18	24	30	12	21	presynapse
F1M8A4	Liprinα	0	0	14	20	7	10	5	7	presynapse
Q9JIR4	Rims1	0	0	10	11	11	12	6	7	presynapse
F1M3B4	UNC13b/MUNC13	0	0	7	8	8	8	0	0	presynapse
R9PY00	Vamp2	0	0	5	5	4	4	0	0	presynapse
P21707	Synaptotagmin1	0	0	4	8	4	6	0	0	presynapse
B5DEH2	Erlin2	1	1	8	9	2	3	0	0	ER
D4ABS2	Dnm1l	0	0	9	10	11	14	2	3	Membrane-related
O08679	Mark2	0	0	19	23	0	0	0	0	kinase
P63086	Mapk1	0	0	8	9	2	2	0	0	kinase
B5DFK6	Ap3d1	0	0	10	16	5	7	0	0	AP-3 complex
P62944	Ap2b1	0	0	15	18	6	9	3	3	AP-2 complex

Table S1. Mass Spectrometry Analysis of KIFC3 Interacting Proteins. Related to Figure 2.

Data from affinity purification mass spectrometry analyses (AP-MS) of KIFC3 and truncations in rat brain extracts. PROTID: Uniprot accession code; Name: corresponding gene name; Peptides: peptide matches; PSM: peptide spectrum matches.

Oligonucleotides	Source	Identifier
**KIFC3 shRNAs and CRISPR KI target sequences**		
pSuper-rat KIFC3 shRNA#1 targeting sequence: cgagaacccaggcattaat	This paper	N/A
pSuper-rat KIFC3 shRNA#2 targeting sequence: cagctccgggacaggttat	This paper	N/A
pSuper-rat KIFC3 shRNA#3 targeting sequence: ccacctgttaagtatgtca	This paper	N/A
pSuper-rat KIFC3 shRNA#4 targeting sequence: ctccctaaatcctatttaa	This paper	N/A
pSuper-rat KIFC3 shRNA#5 targeting sequence: cgcatcagctgacaataca	This paper	N/A
pSuper-mouse KIFC3 shRNA#1 targeting sequence: cagctccgtgacaagctgt	This paper	N/A
pSuper-mouse KIFC3 shRNA#2 targeting sequence: ggcttcaatgtctgtatct	This paper	N/A
pSuper-mouse KIFC3 shRNA#3 targeting sequence: ttccaaactcacctacctg	This paper	N/A
CRISPR KI rat KIFC3 targeting sequence: cggctgcagtttccttcgga	This paper	N/A
**Primers for mouse shRNA resistant KIFC3**		
P1-fw: tcgcggccgcctcgagctcaagcttatggtggaga	This paper	N/A
P1-rev: acagcttgtccctcaattggctgctctcctggctg	This paper	N/A
P2-fw: ccaattgagggacaagctgtcccagctgc	This paper	N/A
P2-rev: ggaacacgtcctgttggcttgcccacggggagaagacc	This paper	N/A
P3-fw: aagccaacaggacgtgttccaggaggtgcaggccctcattacctcctgcatcgatggatttaac	This paper	N/A
P3-rev: gttaaatccatcgatgcaggaggtaatgagggcctgcacctcctggaacacgtcctgttggctt	This paper	N/A
P4-fw: cctgcatcgatggatttaacgtctgtatctttgcttacggc	This paper	N/A
P4-rev: tcagcttgctattgcggaagggcacatg	This paper	N/A
P5-fw: cttccgcaatagcaagctgacctacctgctgcaggac	This paper	N/A
P5-rev: atcccgggcccgcggtaccgtcgactcaggctgacggctgcag	This paper	N/A

Table S2. Oligonucleotides. Related to Oligonucleotides section of the Key Resources Table of the STAR Methods.
