# Supplementary material for: Microtubule Minus-End Binding Protein CAMSAP2 and Kinesin-14 Motor KIFC3 Control Dendritic Microtubule Organization
Source: Curr Biol. 2020 Mar 9;30(5):899–908.e6. doi: 10.1016/j.cub.2019.12.056 (PMC7063570; doi:10.1016/j.cub.2019.12.056)
Supplement: Document S2. Article plus Supplemental Information [file mmc5.pdf]

Report

Current Biology

Microtubule Minus-End Binding Protein CAMSAP2 and Kinesin-14 Motor KIFC3 Control Dendritic Microtubule Organization

Graphical Abstract

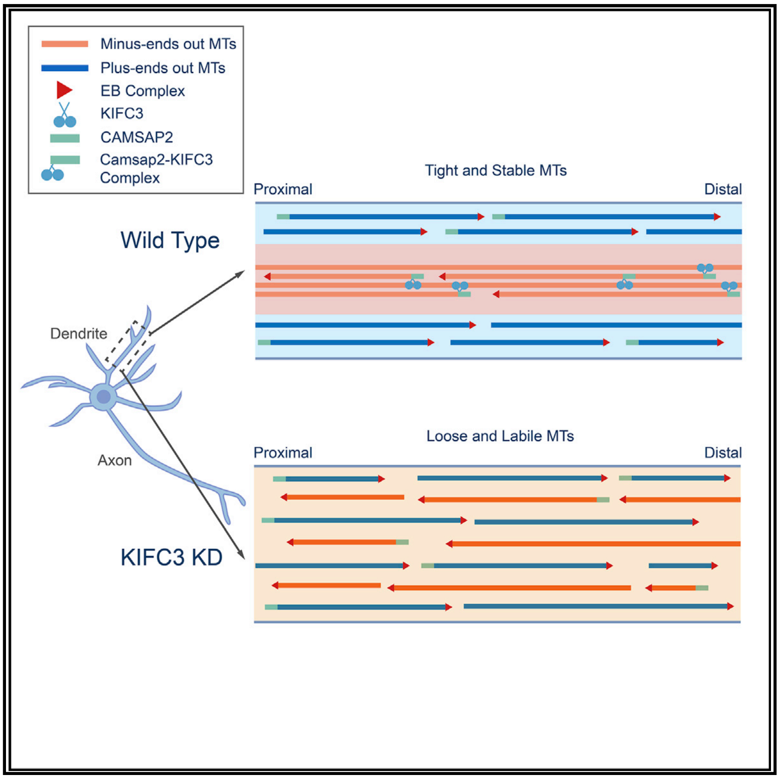

Authors

Yujie Cao, Joanna Lipka,
Riccardo Stucchi, ..., Lukas C. Kapitein,
Martin Harterink,
Casper C. Hoogenraad

Correspondence

c.hoogenraad@uu.nl

In Brief

Neuronal dendrites are characterized by an anti-parallel microtubule organization. The mechanism that regulates dendritic microtubule organization is still unclear. Cao et al. demonstrate that the microtubule minus-end binding protein CAMSAP2 and kinesin-14 motor KIFC3 work together to organize dendritic microtubules and control dendrite branching.

Highlights

- KIFC3 localizes to dendrites and controls dendrite branching
- KIFC3 interacts with minus-end binding protein CAMSAP2
- CAMSAP2 and KIFC3 immobilize microtubule arrays in dendrites

Cao et al., 2020, Current Biology *30*, 899–908
March 9, 2020 © 2020 The Authors. Published by Elsevier Ltd.
https://doi.org/10.1016/j.cub.2019.12.056

CellPress

Microtubule Minus-End Binding Protein CAMSAP2 and Kinesin-14 Motor KIFC3 Control Dendritic Microtubule Organization

Yujie Cao,[1] Joanna Lipka,[1] Riccardo Stucchi,[1,2] Mithila Burute,[1] Xingxiu Pan,[1] Sybren Portegies,[1] Roderick Tas,[1] Jelmer Willems,[1] Lena Will,[1] Harold MacGillavry,[1] Maarten Altelaar,[2] Lukas C. Kapitein,[1] Martin Harterink,[1] and Casper C. Hoogenraad[1,3,4,*]
[1]Cell Biology, Neurobiology and Biophysics, Department of Biology, Faculty of Science, Utrecht University, 3584 Utrecht, the Netherlands
[2]Biomolecular Mass Spectrometry and Proteomics, Bijvoet Center for Biomolecular Research and Utrecht Institute for Pharmaceutical Sciences, Utrecht University, 3584 Utrecht, the Netherlands
[3]Department of Neuroscience, Genentech, Inc., South San Francisco, CA 94080, USA
[4]Lead Contact
*Correspondence: c.hoogenraad@uu.nl
https://doi.org/10.1016/j.cub.2019.12.056

SUMMARY

Neuronal dendrites are characterized by an anti-parallel microtubule organization. The mixed oriented microtubules promote dendrite development and facilitate polarized cargo trafficking; however, the mechanism that regulates dendritic microtubule organization is still unclear. Here, we found that the kinesin-14 motor KIFC3 is important for organizing dendritic microtubules and to control dendrite development. The kinesin-14 motor proteins (*Drosophila melanogaster* Ncd, *Saccharomyces cerevisiae* Kar3, *Saccharomyces pombe* Pkl1, and *Xenopus laevis* XCTK2) are characterized by a C-terminal motor domain and are well described to organize the spindle microtubule during mitosis using an additional microtubule binding site in the N terminus [1–4]. In mammals, there are three kinesin-14 members, KIFC1, KIFC2, and KIFC3. It was recently shown that KIFC1 is important for organizing axonal microtubules in neurons, a process that depends on the two microtubule-interacting domains [5]. Unlike KIFC1, KIFC2 and KIFC3 lack the N-terminal microtubule binding domain and only have one microtubule-interacting domain, the motor domain [6, 7]. Thus, in order to regulate microtubule-microtubule crosslinking or sliding, KIFC2 and KIFC3 need to interact with additional microtubule binding proteins to connect two microtubules. We found that KIFC3 has a dendrite-specific distribution and interacts with microtubule minus-end binding protein CAMSAP2. Depletion of KIFC3 or CAMSAP2 results in increased microtubule dynamics during dendritic development. We propose a model in which CAMSAP2 anchors KIFC3 at microtubule minus ends and immobilizes microtubule arrays in dendrites.

RESULTS

KIFC3 Localizes to the Soma and Dendrites and Controls Dendritic Branching

In order to determine the role of KIFC3 during neuronal development, we depleted endogenous KIFC3 protein from cultured hippocampal neurons at stage 3 with short hairpin RNAs (shRNAs). The efficiency of our shRNAs was quantified by western blot (Figures S1A and S1B). KIFC3 shRNA1, shRNA2, and shRNA4 efficiently reduced KIFC3 protein levels and were subsequently used for the experiments in this study. Depletion of KIFC3 during stage 3 led to simplified dendritic arbors as determined by Sholl analysis (Figures 1A, 1B, and S1C) and shorter total dendrite length (Figure 1C). KIFC3 depletion did not result in significant changes in the number of total dendritic arbors or the average dendrites length (Figures 1D and 1E). We next performed *ex vivo* electroporation experiments to study KIFC3 function in the mouse cortex (Figures S1D and S1E). We found that KIFC3-depleted neurons can still migrate through the cortex but showed abnormal dendrite development (Figure 1F). We defined the cortical neurons into four different categories according to their morphology (Figure 1G). Consistent with the morphology phenotype in primary cultured neurons, the KIFC3 depletion in cortical slices leads to simplified dendritic arbors. The branching defect is rescued by shRNA-resistant KIFC3 expression (Figure 1G). Together, these data suggest that KIFC3 is required for the proper dendrite morphology, but not for neuronal migration.

We next determined the cellular localization of KIFC3. We found that full-length mCherry-tagged KIFC3 decorates microtubule stretches in dendrites and that it is excluded from the axon (Figures 2A and 2D). KIFC3 preferentially co-localizes with stable microtubule bundles, marked by acetylated microtubule arrays, and less with more labile tyrosinated microtubules (Figures 2A–2C). Recent super-resolution data showed that stable and acetylated microtubules are mostly oriented minus end out [8]. Therefore, our data suggest that KIFC3 associates with bundles of mostly minus-end-out-oriented stable microtubules. KIFC3 consists of three N-terminal coiled-coil domains and a C-terminal motor domain (Figure 2G). Because kinesin-14 family

This is an open access article under the CC BY license (http://creativecommons.org/licenses/by/4.0/).

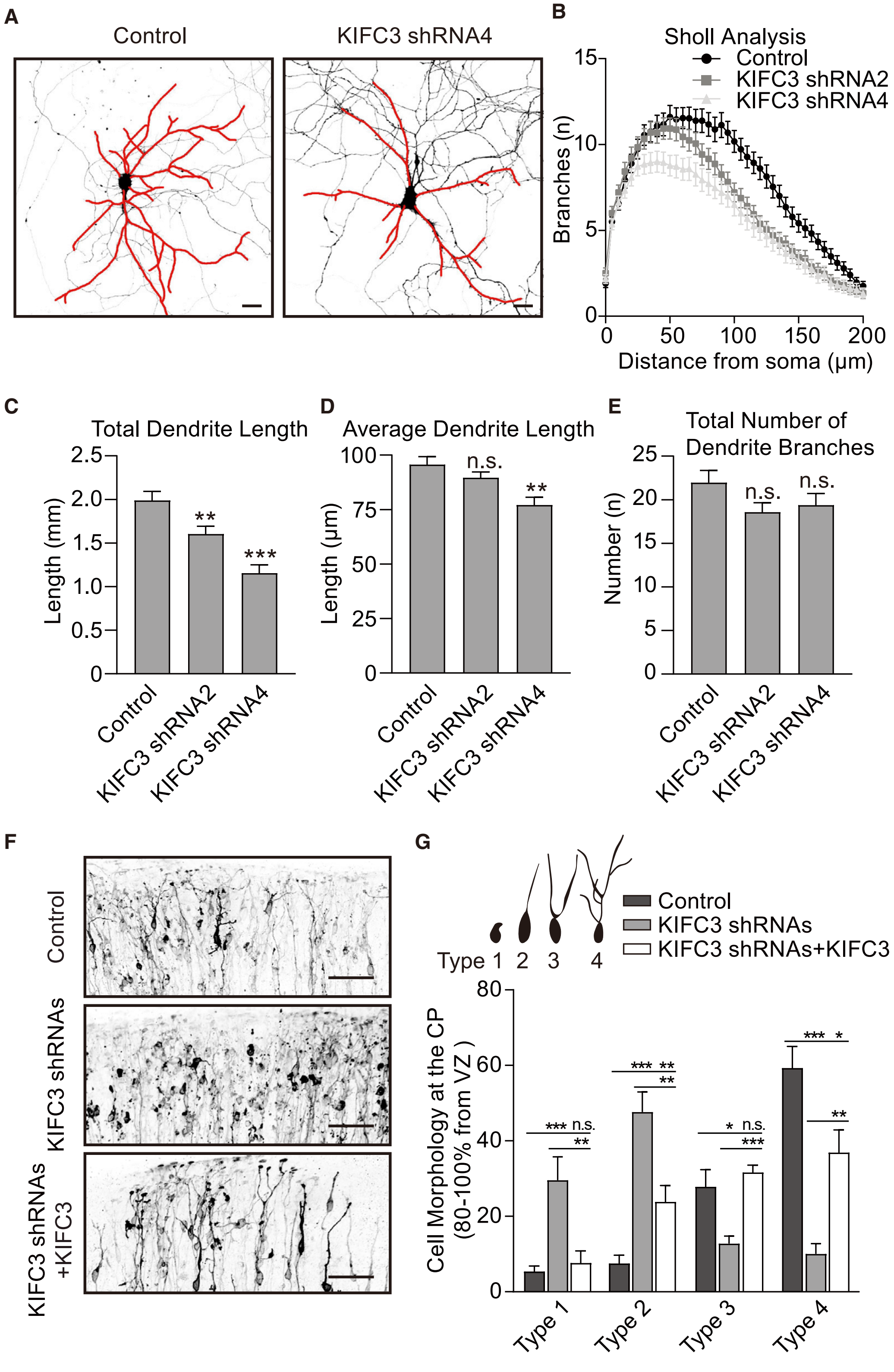

Figure 1. KIFC3 Is Important for Dendrite Branching

(A and B) DIV11 hippocampal neurons expressing GFP and pSuper-scrambled shRNA as a control or KIFC3 shRNAs. shRNA efficiency was indicated in Figures S1A and S1B.

(A) Representative images of the neurons with the dendrites marked in red. Scale bars, 25 μm.

(legend continued on next page)

members are minus-end-directed motors, the axon exclusion of full-length KIFC3 could be the result of retrograde motor activity in the axon toward the cell body. To test this, we generated five truncation mutants that lack the motor domain, from KIFC3-N1 to KIFC3-N5 (Figure 2G). KIFC3-FL (Figure 2D) and KIFC3-N1 (Figure 2E), but not the shorter KIFC3-N2 (Figure 2F) and -N5 (data not shown), localized specifically to the somatodendritic compartment (Figure 2H). These data suggest that the axonal exclusion of KIFC3 is not due to its motor domain. Unfortunately, the anti-KIFC3 antibodies that worked on western blot did not give specific endogenous KIFC3 staining in neuronal cultures. In the next section, we applied the HITI CRISPR knockin system to integrate a tag into the endogenous KIFC3 gene [9].

KIFC3 Interacts with Minus-End Binding Protein CAMSAP2

To investigate the role of KIFC3 in dendrite development, we performed KIFC3 pull-down experiments from brain lysates followed by mass spectrometry to identify binding partners. GFP-tagged KIFC3 full length, KIFC3-N1, and KIFC3-N3 with a BirA recognition site together with the biotin ligase BirA were expressed in HEK293 cells and were used as bait for a pull-down from brain extracts. GFP protein was used as negative control. Several potential KIFC3-associated proteins were identified by mass spectrometry, including several cytoskeleton proteins, such as the microtubule minus-end binding protein CAMSAP2 (Table S1). CAMSAP2 is known to protect and stabilize free microtubule minus ends and is required for neuronal polarity and development [10]. To further validate the interaction between KIFC3 and CAMSAP2, we performed additional pull-down experiments. KIFC3 full length was used as bait and pulled down endogenous CAMSAP2 from HEK293T (Figure 2I). The interaction was not specific to CAMSAP2, because we found that KIFC3 can also pull down the other two CAMSAPs family members (Figure 2J). Expression of full-length GFP-tagged KIFC3 in COS7 cells (Figure S2A) or cultured neurons (Figure S2C) and co-staining for endogenous CAMSAP2 revealed strong overlap between both proteins. The localization of endogenously tagged KIFC3 is very similar to the full-length mCherry-KIFC3 (Figures 2A and 2D) and reveals specific microtubule staining in dendrites (Figure S2D). Endogenous KIFC3 and endogenous CAMSAP2 coincide along microtubule stretches in cultured neurons. Similar result was observed in live neurons co-expressed with mCherry-tagged KIFC3 and 3GFP-tagged CAMSAP2 (Video S1). To identify the minimal domains required for the KIFC3-CAMSAP2 interaction, we performed pull-down experiments using truncated KIFC3 constructs (Figure 2G). Specifically, the second coiled-coil domain of KIFC3 (mCherry-KIFC3-N4) pulls down full-length CAMSAP2 (Figure 2K). Consistently, mCherry-KIFC3-N4 and GFP-CAMSAP2 markedly coincide in COS7 cells (Figures S2E and S2F). On the CAMSAP2 side, the second and third coiled-coil domain (GFP-CAMSAP2-N2 and -N3) co-localized with KIFC3, but not the C terminus or other N-terminal fragments (Figures S2B and S2G–S2J). Altogether, these results suggest that KIFC3 uses its second coiled-coil domain to bind to the second and third coiled-coil domain of CAMSAP2.

As a member of kinesin 14 family, the C-terminal motor domain drives KIFC3 in toward the microtubule minus ends. In this way, CAMSAP2—through its interaction—may capture KIFC3 at the microtubule minus ends. To test this, we performed microtubule-severing experiments in COS7 cell expressing both CAMSAP2 and KIFC3 (Figures 2L and 2M; Video S2). We found that KIFC3 and CAMSAP2 co-localize at new generated microtubule minus ends after cutting. These data suggest that KIFC3 accumulates at microtubule minus ends together with CAMSAP2.

KIFC3 and CAMSAP2 Regulate Microtubule Density and Dynamics in Dendrites

We next tested the role of KIFC3 and CAMSAP2 in dendritic microtubule organization. Depletion of KIFC3 or CAMSAP2 causes a marked reduction in total microtubules and stable and dynamic microtubules, as marked by α-tubulin and acetylated and tyrosinated tubulin, in dendrites of day *in vitro* 11 (DIV11) neurons (Figures 3A–3C), suggesting that both KIFC3 and CAMSAP2 control microtubule density in dendrites. KIFC3 expression also reduced the intensity of tyrosinated tubulin in dendrite but hardly affects the acetylated-tubulin staining (Figure S3A). Next, we determined the dynamics and orientation of dendritic microtubules using the microtubule plus-end marker, GFP-MACF18 (Figure 3D). KIFC3 depletion, using two independent shRNAs, strongly increased the number of retrograde MT+TIP comets tagged by GFP compared to control neurons (Figures 3E and 3F). This effect was rescued by full-length KIFC3, but not KIFC3-CC-MD lacking the CAMSAP2 interacting N-terminal region (Figures 3H and 3I). We next tested whether the KIFC3 motor activity is required for this effect and generated, based on the KIF5B rigor mutation [11], a KIFC3(396T-N) rigor mutant, which cannot walk but still binds to microtubules (Figures S3B and S3C). The KIFC3 rigor mutant did not rescue the KIFC3 knockdown phenotype (Figure 3J), suggesting that the KIFC3 motor activity is required for regulating dendritic microtubule dynamics. The number of retrograde comets was also increased upon CAMSAP2 depletion; however, in contrast to KIFC3 shRNAs, the number of anterograde comets was reduced (Figure 3E), suggesting that CAMSAP2 has additional function in

(B) Sholl analysis of the dendritic branching upon control and KIFC3 depletion. Control: N = 7, n = 46; KIFC3 shRNA2: N = 7, n = 57; KIFC3 shRNA4: N = 5, n = 36. Error bars represent SEM. Significant difference was shown in Figure S1C.

(C–E) Quantification of the neurons described in (A) and (B). Total dendrite length (C), average dendrite length (D), and total number of dendrite branches (E) were quantified. Error bars, SEM. *p < 0.05; **p < 0.01; ***p < 0.001 (unpaired t test).

(F) Representative images of mouse cortical plate. Same images with larger view were used to quantify migration defects (Figures S1D and S1E). Neurons were transfected with MARCKS-GFP and KIFC3 shRNAs or pSuper-scrambled shRNA. A shRNAs-resistant mCherry-KIFC3 was used for rescue. Scale bars, 50 μm. Control: N = 10, n = 175; KIFC3 shRNAs: N = 8, n = 184; KIFC3 shRNAs+KIFC3: N = 9, n = 264.

(G) Quantifications of the neuronal morphology of transfected cells in the cortical plate (CP), defined as 80%–100% of the radial axis from ventricular surface to the pial surface corresponding to (F). Error bars, SEM. *p < 0.05; **p < 0.01; ***p < 0.001 (unpaired t test). Schematic diagram was used to show the typical morphology of different type neurons.

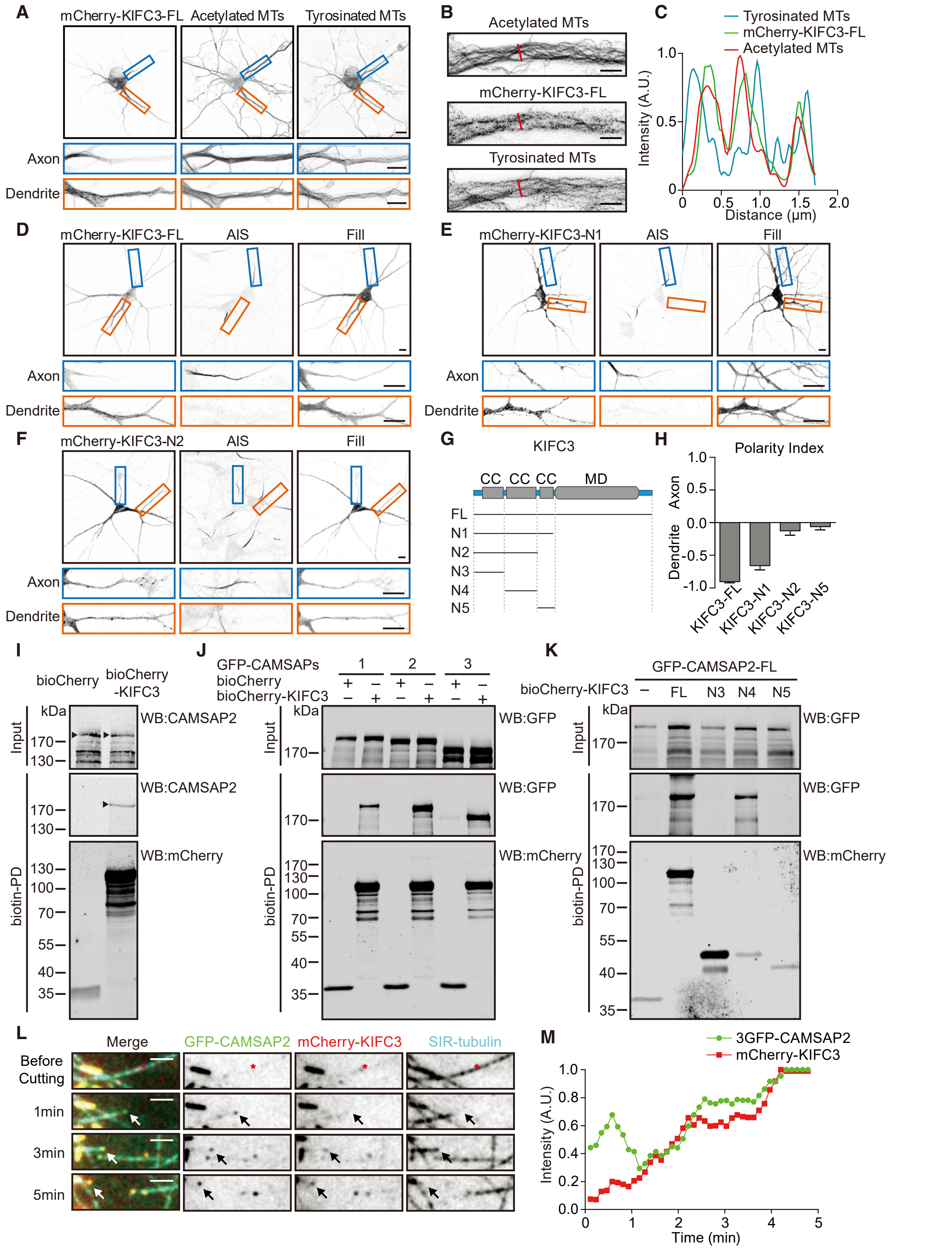

(legend on next page)

dendrites. To assess total microtubule polarity (dynamic and stable), we quantified minus- and plus-end-out microtubules after laser photoablation to generate new plus ends [10]. This showed no difference in microtubule orientation between control and KIFC3- and CAMSAP2-depleted neurons (Figure 3F). To determine whether the KIFC3-CAMSAP2 interaction is important to regulate microtubules dynamics, we generated a CAMSAP2-KIFC3 chimeric construct, which is a direct fusion between the CAMSAP2 minus-end binding domain (CAMSAP-MD-CKK) and the KIFC3, the third coiled-coil, and motor domain (KIFC3-CC-MD; Figure 3G). Although the individual KIFC3-CC-MD and CAMSAP2-MD-CKK constructs were not able to rescue the knockdown phenotype, the chimera fully rescued the microtubules dynamics alterations after KIFC3 depletion (Figure 3I). These results suggest that the KIFC3-CAMSAP2 interaction is required for proper microtubule dynamics in dendrites.

KIFC3 Prevents Microtubule Mobility in Dendrites

We next tested whether KIFC3 and CAMSAP2 control the stability of microtubule bundles in dendrites. Neurons were transfected with photoactivatable GFP-α-tubulin, mCherry-α-tubulin, and either control, KIFC3, or CAMSAP2 shRNA. Segments of 15 μm in the middle of the dendrites were photoactivated and imaged over 3 h (Figures 4A and 4C; Video S3). In wild-type neurons, retrograde displacement of the photoconverted region was observed; only ~25% of the neurons displayed microtubule bundle elongation (longer than 0.8-μm elongation) of the photoconverted region (Figure 4B). However, upon KIFC3 or CAMSAP2 knockdown, the dendritic microtubule bundles became dynamic; in ~80% of the neurons, the photoactivated region elongated and moved toward the soma (Figures 4A–4C and S3D–S3J). The microtubule displacement can be rescued by KIFC3 or KIFC3-CAMSAP2 chimera expression (Figure 4D). These data suggest that the interaction between KIFC3 and CAMSAP2 immobilizes microtubule arrays in dendrites (Figure 4E). We next tested the KIFC3-rigor mutant and microtubule stabilization agent Taxol treatment in these experiments. Interestingly, both conditions could rescue the displacement phenotype (Figure 4D), suggesting KIFC3 motor activity is not required for microtubule crosslinking and bundling. All together, these results suggest KIFC3 and CAMSAP2 are important for microtubule stabilization and immobilization in dendrites.

DISCUSSION

In epithelial cells, KIFC3 was found to localize to the zonula adherens (ZA), a specialized cadherin-based structure found at the contacts between cells [12]. The localization of KIFC3 depends upon pleckstrin-homology-domain-containing family A member 7 (PLEKHA7), CAMSAP3, and microtubules. Depletion of any of these factors resulted in disorganized ZA, suggesting that KIFC3 may provide a structural link between microtubules and the ZA at epithelial cell-cell junctions [12]. Alternatively, KIFC3 may play a role in cargo transport required for ZA formation. Consistent with a role in trafficking in epithelial cells, KIFC3 has also been described to transport annexin XIIIb to the apical membranes and the ubiquitin-specific protease 47 (USP47) to the adherens junction [13, 14]. Moreover, KIFC3 has been postulated to participate in positioning of the Golgi apparatus under cholesterol-depleted conditions [15]. Here, we found that KIFC3 in neurons has a more structural role by working together with minus-end binding protein CAMSAP2 to organize dendritic microtubules.

The CAMSAP/Patronin/Nezha family has been characterized previously and found to specifically recognize microtubule minus ends and stabilize microtubules against depolymerization [16]. In *Drosophila*, the CAMSAP family member, named Patronin, associates with free microtubule minus ends and inhibits their disassembly by the action of the kinesin-13 microtubule depolymerase [17]. In mammals, all three CAMSAP family members recognize growing microtubule minus ends, and CAMSAP2 and CAMSAP3 form stretches that are stably deposited on the MT lattice generated by minus-end polymerization [18, 19]. In worms, loss of the CAMSAP homolog PTRN-1 caused alterations in neuronal morphology and synaptic vesicle localization

Figure 2. KIFC3 Localizes to Dendrites and Interacts with CAMSAP2

(A and B) Representative images of DIV11 hippocampal neurons transfected with mCherry-KIFC3-FL and co-stained with acetylated tubulin and tyrosinated tubulin antibody.

(A) Selected axonal (marked with a blue box) and dendritic (marked with an orange box) regions are enlarged below. Scale bars, 10 μm and 5 μm in enlargements.

(B) Further enlargement of a dendrite region is shown. Scale bars, 2 μm.

(C) Intensity profile of the indicated region in (B).

(D–F) Representative images of DIV10 hippocampal neurons transfected with GFP fill and mCherry-KIFC3-FL (D), mCherry-KIFC3-N1 (E), or mCherry-KIFC3-N2 (F) and stained for TRIM46 to visualize the axon initial segment (AIS). Selected axonal (marked with a blue box) and dendritic (marked with an orange box) regions are enlarged below. Scale bars, 10 μm.

(G) Schematic representation of the KIFC3 secondary structure and truncation constructs used. CC, coiled-coiled domain; MD, motor domain.

(H) Polarity index analysis from KIFC3-FL and truncations corresponding to (D)–(F). Positive values indicate axon enrichment, and negative values indicate dendrite enrichment. Error bars, SEM. N = 2. KIFC3-FL: n = 12, KIFC3-N1: n = 10, KIFC3-N2: n = 9, and KIFC3-N5: n = 6.

(I) Biotin pull-downs from extracts of HEK293T cells transfected with BirA recognition site conjugated mCherry-KIFC3-FL and probed for mCherry and CAMSAP2. For all pull-down experiments, the input is 25% of the biotin pull-down. CAMSAP2 was found from KIFC3 pull-downs with brain extracts (Table S1). The interaction is also proved by co-localization experiment in neurons and COS7 cells (Figure S2; Video S1).

(J) Biotin pull-downs from extracts of HEK293T cells transfected with BirA recognition site conjugated mCherry-KIFC3-FL together with GFP-tagged CAMSAP1, 2, or 3 and probed for mCherry and GFP.

(K) Biotin pull-downs from extracts of HEK293T cells transfected with BirA recognition site conjugated KIFC3 truncation constructs together with GFP-tagged CAMSAP2 and probed for mCherry and GFP.

(L) Representative images of COS7, transfected with 3GFP-CAMSAP2 and mCherry-KIFC3 (Video S2). Silicon rhodamine (SIR)-tubulin was applied to visualize microtubules. Microtubule photoablation was indicated by red stars. Minus end was indicated by white and black arrows. 3GFP-CAMSAP2 was shown in green, mCherry-KIFC3 in red, and SIR-tubulin in cyan. Scale bars, 2 μm.

(M) The quantification of CAMSAP2 and KIFC3 puncta intensity corresponding to (L).

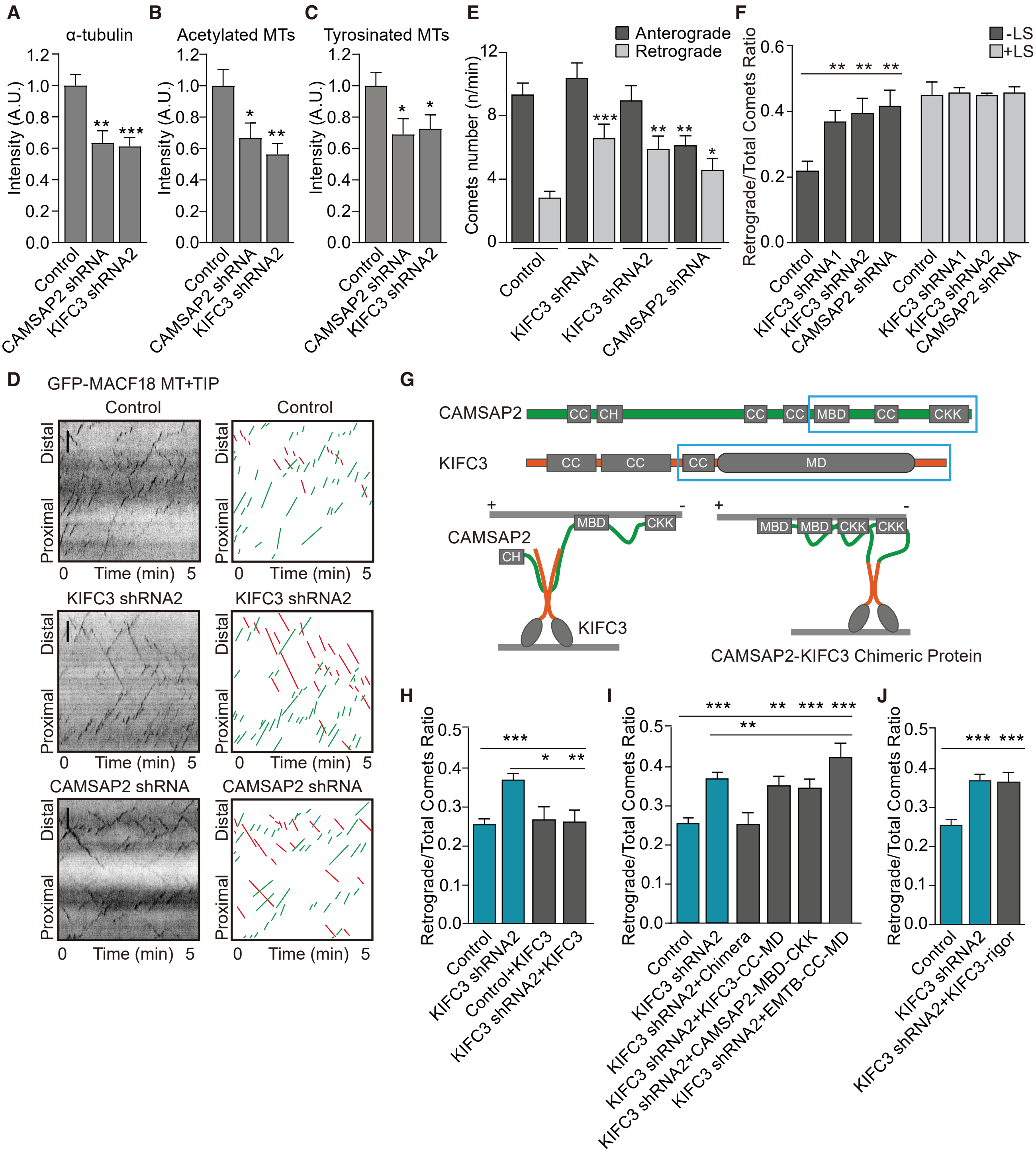

Figure 3. CAMSAP2 and KIFC3 Stabilize Minus-End-Out Microtubules

(A–C) Quantification of α-tubulin (A), acetylated tubulin (B), and tyrosinated tubulin (C) levels in DIV11 neurons transfected with pSuper scrambled, CAMSAP2 shRNA, KIFC3 shRNA2, or KIFC3 overexpression (Figure S3A). (A) Control: N = 7, n = 58; CAMSAP2 shRNA: N = 7, n = 25; KIFC3 shRNA2: N = 7, n = 34. (B) Control: N = 3, n = 25; CAMSAP2 shRNA: N = 3, n = 12; KIFC3 shRNA2: N = 3, n = 15. (C) Control: N = 3, n = 25; CAMSAP2 shRNA: N = 3, n = 9; KIFC3 shRNA2: N = 3, n = 16. Error bars, SEM. Unpaired t test was performed, and columns were compared with corresponding control. *p < 0.05; **p < 0.01; ***p < 0.001.

(D) Original and illustrated kymographs of GFP-MACF18 traced with life-cell microscopy from control and KIFC3-shRNA2- and CAMSAP2-shRNA-transfected neurons. GFP-MACF18 was used to visualize microtubule plus-end tips. Anterograde comets are marked in green, and retrograde comets are marked in red. Scale bars, 3 μm.

(E) Quantification of the number of comets moving retrogradely and anterogradely in dendrites of neurons described in (C). N = 2, control: n = 26, KIFC3 shRNA1: n = 20, KIFC3 shRNA2: n = 19, CAMSAP2 shRNA: n = 20. Error bars, SEM. *p < 0.05; **p < 0.01; ***p < 0.001 (unpaired t test).

(legend continued on next page)

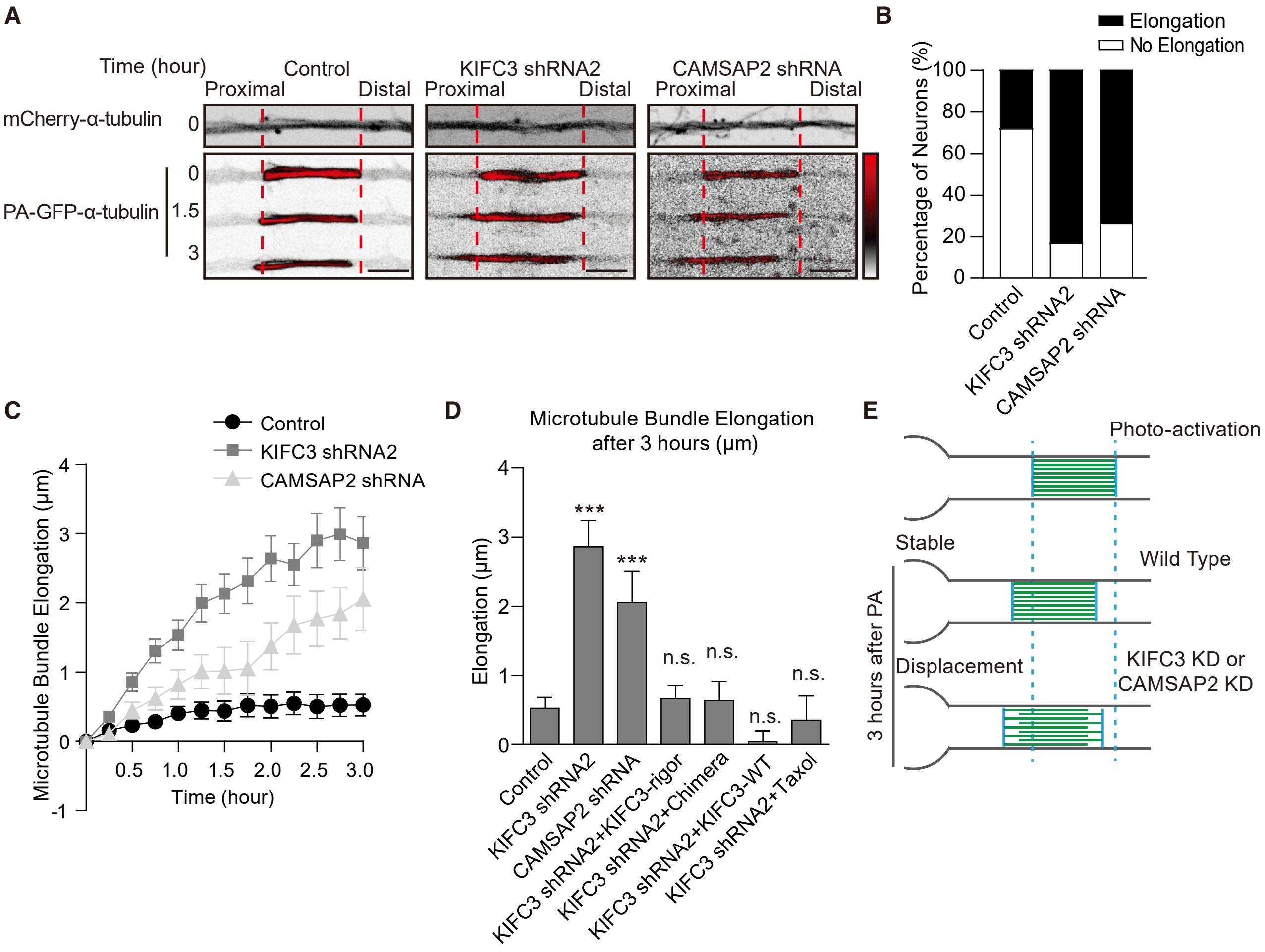

Figure 4. Dendritic Microtubules Become More Dynamic with KIFC3 or CAMSAP2 Depletion

(A) Representative images of dendrites of hippocampal neurons DIV12 transfected with mCherry-α-tubulin, photoactivated GFP-α-tubulin together with pSuper-scrambled control, KIFC3 shRNA2, or CAMSAP2 shRNA (Video S3). Time is indicated at the left of each image. PA-GFP channel was indicated in black and red, and LUT was shown from the right. Red dash lines indicate the photoactivated microtubule region. Scale bars, 5 µm.

(B) Quantification of the percentage of the neurons described in (A), in which the photoconverted region elongated.

(C) Quantification of microtubule bundle elongation. Other measurements were shown in Figures S3D–S3J. Control: N = 5, n = 36; KIFC3 shRNA2: N = 4, n = 35; CAMSAP2 shRNA: N = 2, n = 15. Error bars, SEM.

(D) Quantification of microtubule bundle elongation 3 h after photo-conversion corresponding to (A) and (C). KIFC3 shRNA2+KIFC3-rigor: N = 2, n = 20; KIFC3 shRNA2+KIFC3-Chimera: N = 3, n = 10; KIFC3 shRNA2+KIFC3-WT: N = 3, n = 12; KIFC3 shRNA2+Taxol: N = 3, n = 8. Error bars, SEM. Columns were compared with control. *$p < 0.05$; **$p < 0.01$; ***$p < 0.001$ (unpaired t test).

(E) Schematic graph of microtubule displacement in (A).

[20–22] and also affected the axonal regeneration after injury [23]. In mammalian neurons, CAMSAP may stabilize short-microtubule stretches to serve as seeds for microtubule regrowth [10]. We propose a model in which CAMSAP2 anchors KIFC3 at microtubule minus ends and immobilizes microtubule arrays in dendrites.

(F) Quantification of the ratio of retrograde/total comets in dendrites of cells described in (D) with (black bars) and without (white bars) laser severing. No laser severing: N = 2, control: n = 26, KIFC3 shRNA1: n = 20, KIFC3 shRNA2: n = 19, CAMSAP2 shRNA: n = 20. Conditions with laser severing: control N = 1, n = 4; KIFC3 shRNA1 N = 1, n = 3; KIFC3 shRNA2 N = 1, n = 3; CAMSAP2 shRNA N = 2, n = 3. Error bars, SEM. *$p < 0.05$; **$p < 0.01$; ***$p < 0.001$ (unpaired t test).

(G) Schematic representation of the KIFC3, CAMSAP2, and CAMSAP2-KIFC3 chimeric protein. CH, calponin-homology domain; CKK, CKK domain; MBD, microtubule binding domain. The domains present in the chimeric protein are marked by blue rectangles. Green lines mark amino acids from CAMSAP2; orange lines mark amino acids from KIFC3. Microtubules with their orientations are marked in gray.

(H–J) Quantification of retrograde/total comets ratio in dendrites of neurons transfected with pSuper-scrambled as control or KIFC3 shRNA2 together with different KIFC3 rescue constructs. KIFC3-rigor was validated by peroxisome distribution assay (Figures S3B and S3C). Control: N = 12, n = 78; KIFC3 shRNA2: N = 13, n = 85; control+KIFC3-FL: N = 3, n = 13; KIFC3 shRNA2+KIFC3-FL: N = 3, n = 21; KIFC3 shRNA2+Chimera: N = 3, n = 18; KIFC3 shRNA2+KIFC3-CC-MD: N = 3, n = 22; KIFC3 shRNA2+CAMSAP2-MBD-CKK: N = 4, n = 37; KIFC3 shRNA2+EMTB-MBD-CKK: N = 3, n = 15; KIFC3 shRNA2+KIFC3-rigor: N = 4, n = 32. Error bars, SEM. Columns were compared with control or KIFC3 shRNA2, respectively. *$p < 0.05$; **$p < 0.01$; ***$p < 0.001$ (unpaired t test).

KIFC1 has been found to slide microtubule during cell division [24]. *In vitro*, KIFC1 could form bundles and aster shape microtubules by joining microtubule minus ends together and create higher order microtubule organization of the axonal microtubule [6]. In neurons, KIFC1 was found to organize axonal microtubules using a second microtubule binding domain [5]. Unlike KIFC1, KIFC3 does not have the second microtubule binding domain required for crosslinking microtubules. The KIFC3 association with CAMSAP2 may provide the additional microtubule binding site to form microtubule bundles. Recently, it was suggested that KIFC3 may form a tetrameric kinesin through its tail interactions *in vitro* [25]. The potential interaction between tetrameric KIFC3 and CAMSAP2 is not inconsistent with our model. The KIFC3-rigor mutation was not able to rescue the KIFC3 knockdown phenotype, suggesting that KIFC3 motor activity is important for dendritic microtubule organization. The force generated by the KIFC3 motor domain may facilitate bundle formation and crosslink microtubules together. Microtubule bundling and crosslinking by the KIFC3-CAMSAP2 interaction could prevent microtubule sliding driven by other motor proteins. This model is consistent with the observation that microtubule mobility and sliding was increased in KIFC3- and CAMSAP2-depleted neuron.

Recent super-resolution experiments suggest that microtubules with the same orientation are bundled in dendrites [8]. Stable and acetylated microtubules are mostly oriented minus-end out, although dynamic and tyrosinated microtubules are oriented oppositely. We found that KIFC3 prefers acetylated microtubules in dendrites, which suggests KIFC3 may recognize minus-end-out microtubules. It is tempting to speculate that the KIFC3-CAMSAP2 interaction may preferentially stabilize minus-end-out microtubules in dendrite. Consistently, in KIFC3-depleted neurons, microtubule acetylation was decreased and minus-end-out microtubules showed increased dynamics. Some kinesins are reported to be directly sensitive for microtubule modifications, which may influence their activity in neurons [26, 27]. However, building a coherent model of how microtubule modifications influence KIFC3 function and microtubule organization will require additional work.

Together, our data provide new insights in dendritic microtubule organization. We propose a model in which CAMSAP2 anchors KIFC3 at microtubule minus ends to immobilize microtubule arrays in dendrites. Parallel mechanisms could play additional roles in organizing microtubule dynamics. For example, recently, APC2, the brain-specific homolog of tumor-suppressor protein adenomatous polyposis coli (APC), has been shown to promote minus-end-out microtubule dynamics in dendrites [28]. Recent developments in super-resolution and single-molecule imaging techniques could be applied to further investigate neuronal microtubule structure.

STAR★METHODS

Detailed methods are provided in the online version of this paper and include the following:

- KEY RESOURCES TABLE
- LEAD CONTACT AND MATERIALS AVAILABILITY
- EXPERIMENTAL MODEL AND SUBJECT DETAILS
  - Animals
  - Cell line culture and transfection
  - Primary neuron culture and transfection
  - *Ex vivo* electroporation and organotypic brain slice cultures
- METHOD DETAILS
  - DNA and shRNA constructs
  - Antibody and reagents
  - Biotin-streptavidin pull-down and western blot
  - Biotin-streptavidin pull-down and Mass spectrometry
  - Immunofluorescence fixation and staining
  - Slice immunofluorescence and imaging
  - Laser Scanning Confocal and STED microscopy
  - Live cell imaging
- QUANTIFICATION AND STATISTICAL ANALYSIS
  - Sholl analysis
  - Quantification of *ex vivo* neuronal migration and morphology
  - Mass spectrometry analysis
  - Analysis of polarity index
  - Microtubule ablation in COS7
  - PEX quantification
- DATA AND CODE AVAILABILITY

SUPPLEMENTAL INFORMATION

Supplemental Information can be found online at https://doi.org/10.1016/j.cub.2019.12.056.

ACKNOWLEDGMENTS

We thank Dr. Anna Akhmanova for sharing bio-mCherry and bio-GFP-N1 constructs. This work was supported by the Netherlands Organization for Scientific Research (NWO-ALW-VICI; 865.10.010; C.C.H.), the Netherlands Organization for Health Research and Development (ZonMW-TOP; 912.16.058; C.C.H.), the European Research Council (ERC) (ERC-consolidator; 617050; C.C.H.), and China Scholarship Council (CSC).

AUTHOR CONTRIBUTIONS

Y.C. designed and performed the experiments, analyzed results, and wrote the manuscript; J.L. initiated the project and designed KIFC3 shRNAs; R.S. performed the mass spectrometry experiment; R.T. helped with super-resolution imaging; M.B. helped with the microtubule-severing experiment in COS7 and PEX imaging and quantification; L.W. and S.P. performed *ex vivo* electroporation and organotypic slice cultures; X.P. performed immunohistochemistry staining, imaging, and quantification for *ex vivo* experiments; J.W. helped with the CRISPR KI plasmid cloning; H.M., M.A., and L.C.K. gave valuable advice; and M.H. and C.C.H. designed the experiments, supervised the research, and wrote the manuscript.

DECLARATION OF INTERESTS

C.C.H. is an employee of Genentech, a member of the Roche group. The authors declare no additional competing interests.

Received: April 29, 2019
Revised: October 28, 2019
Accepted: December 18, 2019
Published: February 20, 2020

REFERENCES

1. Hirokawa, N. (1998). Kinesin and dynein superfamily proteins and the mechanism of organelle transport. Science *279*, 519–526.
2. Peterman, E.J., and Scholey, J.M. (2009). Mitotic microtubule crosslinkers: insights from mechanistic studies. Curr. Biol. *19*, R1089–R1094.
3. Fink, G., Hajdo, L., Skowronek, K.J., Reuther, C., Kasprzak, A.A., and Diez, S. (2009). The mitotic kinesin-14 Ncd drives directional microtubule-microtubule sliding. Nat. Cell Biol. *11*, 717–723.
4. Braun, M., Drummond, D.R., Cross, R.A., and McAinsh, A.D. (2009). The kinesin-14 Klp2 organizes microtubules into parallel bundles by an ATP-dependent sorting mechanism. Nat. Cell Biol. *11*, 724–730.
5. Muralidharan, H., and Baas, P.W. (2019). Mitotic motor KIFC1 is an organizer of microtubules in the axon. J. Neurosci. *39*, 3792–3811.
6. Norris, S.R., Jung, S., Singh, P., Strothman, C.E., Erwin, A.L., Ohi, M.D., Zanic, M., and Ohi, R. (2018). Microtubule minus-end aster organization is driven by processive HSET-tubulin clusters. Nat. Commun. *9*, 2659.
7. Zhang, Y., and Sperry, A.O. (2004). Comparative analysis of two C-terminal kinesin motor proteins: KIFC1 and KIFC5A. Cell Motil. Cytoskeleton *58*, 213–230.
8. Tas, R.P., Chazeau, A., Cloin, B.M.C., Lambers, M.L.A., Hoogenraad, C.C., and Kapitein, L.C. (2017). Differentiation between oppositely oriented microtubules controls polarized neuronal transport. Neuron *96*, 1264–1271.e5.
9. Suzuki, K., Tsunekawa, Y., Hernandez-Benitez, R., Wu, J., Zhu, J., Kim, E.J., Hatanaka, F., Yamamoto, M., Araoka, T., Li, Z., et al. (2016). In vivo genome editing via CRISPR/Cas9 mediated homology-independent targeted integration. Nature *540*, 144–149.
10. Yau, K.W., van Beuningen, S.F., Cunha-Ferreira, I., Cloin, B.M., van Battum, E.Y., Will, L., Schätzle, P., Tas, R.P., van Krugten, J., Katrukha, E.A., et al. (2014). Microtubule minus-end binding protein CAMSAP2 controls axon specification and dendrite development. Neuron *82*, 1058–1073.
11. Nakata, T., and Hirokawa, N. (1995). Point mutation of adenosine triphosphate-binding motif generated rigor kinesin that selectively blocks anterograde lysosome membrane transport. J. Cell Biol. *131*, 1039–1053.
12. Meng, W., Mushika, Y., Ichii, T., and Takeichi, M. (2008). Anchorage of microtubule minus ends to adherens junctions regulates epithelial cell-cell contacts. Cell *135*, 948–959.
13. Sako-Kubota, K., Tanaka, N., Nagae, S., Meng, W., and Takeichi, M. (2014). Minus end-directed motor KIFC3 suppresses E-cadherin degradation by recruiting USP47 to adherens junctions. Mol. Biol. Cell *25*, 3851–3860.
14. Noda, Y., Okada, Y., Saito, N., Setou, M., Xu, Y., Zhang, Z., and Hirokawa, N. (2001). KIFC3, a microtubule minus end-directed motor for the apical transport of annexin XIIIb-associated Triton-insoluble membranes. J. Cell Biol. *155*, 77–88.
15. Xu, Y., Takeda, S., Nakata, T., Noda, Y., Tanaka, Y., and Hirokawa, N. (2002). Role of KIFC3 motor protein in Golgi positioning and integration. J. Cell Biol. *158*, 293–303.
16. Akhmanova, A., and Hoogenraad, C.C. (2015). Microtubule minus-end-targeting proteins. Curr. Biol. *25*, R162–R171.
17. Goodwin, S.S., and Vale, R.D. (2010). Patronin regulates the microtubule network by protecting microtubule minus ends. Cell *143*, 263–274.
18. Hendershott, M.C., and Vale, R.D. (2014). Regulation of microtubule minus-end dynamics by CAMSAPs and Patronin. Proc. Natl. Acad. Sci. USA *111*, 5860–5865.
19. Jiang, K., Hua, S., Mohan, R., Grigoriev, I., Yau, K.W., Liu, Q., Katrukha, E.A., Altelaar, A.F., Heck, A.J., Hoogenraad, C.C., and Akhmanova, A. (2014). Microtubule minus-end stabilization by polymerization-driven CAMSAP deposition. Dev. Cell *28*, 295–309.
20. Marcette, J.D., Chen, J.J., and Nonet, M.L. (2014). The Caenorhabditis elegans microtubule minus-end binding homolog PTRN-1 stabilizes synapses and neurites. eLife *3*, e01637.
21. Richardson, C.E., Spilker, K.A., Cueva, J.G., Perrino, J., Goodman, M.B., and Shen, K. (2014). PTRN-1, a microtubule minus end-binding CAMSAP homolog, promotes microtubule function in Caenorhabditis elegans neurons. eLife *3*, e01498.
22. Borgen, M.A., Giles, A.C., Wang, D., and Grill, B. (2019). Synapse maintenance is impacted by ATAT-2 tubulin acetyltransferase activity and the RPM-1 signaling hub. eLife *8*, e44040.
23. Chuang, M., Goncharov, A., Wang, S., Oegema, K., Jin, Y., and Chisholm, A.D. (2014). The microtubule minus-end-binding protein patronin/PTRN-1 is required for axon regeneration in C. elegans. Cell Rep. *9*, 874–883.
24. She, Z.Y., and Yang, W.X. (2017). Molecular mechanisms of kinesin-14 motors in spindle assembly and chromosome segregation. J. Cell Sci. *130*, 2097–2110.
25. Hata, S., Pastor Peidro, A., Panic, M., Liu, P., Atorino, E., Funaya, C., Jäkle, U., Pereira, G., and Schiebel, E. (2019). The balance between KIFC3 and EG5 tetrameric kinesins controls the onset of mitotic spindle assembly. Nat. Cell Biol. *21*, 1138–1151.
26. Ikegami, K., Heier, R.L., Taruishi, M., Takagi, H., Mukai, M., Shimma, S., Taira, S., Hatanaka, K., Morone, N., Yao, I., et al. (2007). Loss of alpha-tubulin polyglutamylation in ROSA22 mice is associated with abnormal targeting of KIF1A and modulated synaptic function. Proc. Natl. Acad. Sci. USA *104*, 3213–3218.
27. Sirajuddin, M., Rice, L.M., and Vale, R.D. (2014). Regulation of microtubule motors by tubulin isotypes and post-translational modifications. Nat. Cell Biol. *16*, 335–344.
28. Kahn, O.I., Schätzle, P., van de Willige, D., Tas, R.P., Lindhout, F.W., Portegies, S., Kapitein, L.C., and Hoogenraad, C.C. (2018). APC2 controls dendrite development by promoting microtubule dynamics. Nat. Commun. *9*, 2773.
29. van Beuningen, S.F.B., Will, L., Harterink, M., Chazeau, A., van Battum, E.Y., Frias, C.P., Franker, M.A.M., Katrukha, E.A., Stucchi, R., Vocking, K., et al. (2015). TRIM46 controls neuronal polarity and axon specification by driving the formation of parallel microtubule arrays. Neuron *88*, 1208–1226.
30. Wu, J., de Heus, C., Liu, Q., Bouchet, B.P., Noordstra, I., Jiang, K., Hua, S., Martin, M., Yang, C., Grigoriev, I., et al. (2016). Molecular pathway of microtubule organization at the Golgi apparatus. Dev. Cell *39*, 44–60.
31. Jiang, K., Toedt, G., Montenegro Gouveia, S., Davey, N.E., Hua, S., van der Vaart, B., Grigoriev, I., Larsen, J., Pedersen, L.B., Bezstarosti, K., et al. (2012). A proteome-wide screen for mammalian SxIP motif-containing microtubule plus-end tracking proteins. Curr. Biol. *22*, 1800–1807.
32. Kapitein, L.C., Schlager, M.A., Kuijpers, M., Wulf, P.S., van Spronsen, M., MacKintosh, F.C., and Hoogenraad, C.C. (2010). Mixed microtubules steer dynein-driven cargo transport into dendrites. Curr. Biol. *20*, 290–299.
33. Brummelkamp, T.R., Bernards, R., and Agami, R. (2002). A system for stable expression of short interfering RNAs in mammalian cells. Science *296*, 550–553.
34. Kapitein, L.C., Schlager, M.A., van der Zwan, W.A., Wulf, P.S., Keijzer, N., and Hoogenraad, C.C. (2010). Probing intracellular motor protein activity using an inducible cargo trafficking assay. Biophys. J. *99*, 2143–2152.
35. Lansbergen, G., Grigoriev, I., Mimori-Kiyosue, Y., Ohtsuka, T., Higa, S., Kitajima, I., Demmers, J., Galjart, N., Houtsmuller, A.B., Grosveld, F., and Akhmanova, A. (2006). CLASPs attach microtubule plus ends to the cell cortex through a complex with LL5beta. Dev. Cell *11*, 21–32.
36. Meijering, E., Jacob, M., Sarria, J.C., Steiner, P., Hirling, H., and Unser, M. (2004). Design and validation of a tool for neurite tracing and analysis in fluorescence microscopy images. Cytometry A *58*, 167–176.
37. Schätzle, P., Kapitein, L.C., and Hoogenraad, C.C. (2016). Live imaging of microtubule dynamics in organotypic hippocampal slice cultures. Methods Cell Biol. *131*, 107–126.

38. Kuijpers, M., van de Willige, D., Freal, A., Chazeau, A., Franker, M.A., Hofenk, J., Rodrigues, R.J., Kapitein, L.C., Akhmanova, A., Jaarsma, D., and Hoogenraad, C.C. (2016). Dynein regulator NDEL1 controls polarized cargo transport at the axon initial segment. Neuron *89*, 461–471.

39. Miller, A.L., and Bement, W.M. (2009). Regulation of cytokinesis by Rho GTPase flux. Nat. Cell Biol. *11*, 71–77.

40. Kevenaar, J.T., Bianchi, S., van Spronsen, M., Olieric, N., Lipka, J., Frias, C.P., Mikhaylova, M., Harterink, M., Keijzer, N., Wulf, P.S., et al. (2016). Kinesin-binding protein controls microtubule dynamics and cargo trafficking by regulating kinesin motor activity. Curr. Biol. *26*, 849–861.

41. Cunha-Ferreira, I., Chazeau, A., Buijs, R.R., Stucchi, R., Will, L., Pan, X., Adolfs, Y., van der Meer, C., Wolthuis, J.C., Kahn, O.I., et al. (2018). The HAUS complex is a key regulator of non-centrosomal microtubule organization during neuronal development. Cell Rep. *24*, 791–800.

42. Chazeau, A., Katrukha, E.A., Hoogenraad, C.C., and Kapitein, L.C. (2016). Studying neuronal microtubule organization and microtubule-associated proteins using single molecule localization microscopy. Methods Cell Biol. *131*, 127–149.

STAR★METHODS

KEY RESOURCES TABLE

REAGENT or RESOURCE	SOURCE	IDENTIFIER
Antibodies		
Mouse anti-α-tubulin	Sigma-Aldrich	Cat# T5168; RRID: AB_477579
Mouse anti-Acetylated tubulin	Sigma-Aldrich	Cat# T7451; RRID: AB_609894
Rat anti-Tyrosinated tubulin	Abcam	Cat# Ab6160; RRID: AB_305328
Chicken anti-MAP2	Abcam	Cat# AB5392; RRID: AB_2138153
Chicken anti-GFP	Aves Labs	Cat# GFP-1020; RRID: AB_10000240
Rabbit anti-CAMSAP2	Proteintech	Cat# 17880-1-AP; RRID: AB_2068826
Rabbit anti-KIFC3	Santa Cruz	Cat# sc-134681; RRID: AB_10610803
Rabbit anti-TRIM46	[29]	N/A
Mouse anti-MAP2	Sigma-Aldrich	Cat# M9942; RRID: AB_477256
Rabbit anti-mCherry/RFP	Rockland	Cat# 600-401-379; RRID: AB_2209751
Mouse anti-mCherry	Clonetech	Cat# 632543; RRID: AB_2307319
Rabbit anti-GFP	Abcam	Cat# AB290; RRID: AB_2313768
anti-chicken Alexa405	Abcam	Cat# AB175675; RRID: AB_2810980
anti-rat Alexa488	Life Technologies	Cat# A11006; RRID: AB_2534074
anti-mouse Alexa488	Life Technologies	Cat# A11029; RRID: AB_2534088
anti-rabbit Alexa488	Life Technologies	Cat# A11034; RRID: AB_2576217
anti-chicken Alexa488	Thermo Fisher	Cat# A-11039; RRID: AB_142924
anti-mouse Alexa568	Life Technologies	Cat# A11031; RRID: AB_144696
anti-rabbit Alexa568	Life Technologies	Cat# A11036; RRID: AB_10563566
anti-rabbit Alexa647	Life Technologies	Cat# A21245; RRID: AB_2535813
anti-mouse Alexa647	Life Technologies	Cat# A21236; RRID: AB_2535805
Goat anti-rabbit IgG Antibody, IRDye 680LT Conjugated	LI-COR Biosciences	Cat# 827-11081; RRID: AB_10795015
Goat anti-mouse IgG Antibody, IRDye 680LT Conjugated	LI-COR Biosciences	Cat# 827-11080; RRID: AB_10795014
Goat anti-rabbit IgG Antibody, IRDye 800CW Conjugated	LI-COR Biosciences	Cat# 827-08365; RRID: AB_10796098
Goat anti-mouse IgG Antibody, IRDye 800CW Conjugated	LI-COR Biosciences	Cat# 827-08364; RRID: AB_10793856
Chemicals		
FuGENE 6	Roche	Cat# 11836145001
Lipofectamine 2000	Thermo Fisher	Cat# 11668019
Vectashield mounting medium	Vectorlabs	Cat# H-1000
Doxycycline	Sigma-Aldrich	Cat# D1822
PEI	PolySciences	Cat# 24765–2
Rapalog	Takara	Cat# 635057
SIR-tubulin	Spiro Chrome	Cat# SC002
Critical Commercial Assays		
Rat Neuron Nucleofector kit	Amaxa	Cat# VVPG-1003
Dynabeads M-280 streptavidin beads	Thermo Fisher	Cat# 11206D
Experimental Models: Cell Lines		
African Green Monkey SV40-transformed kidney ATCC CRL-1651 fibroblast (COS-7)	ATCC	Cat# CRL-1651
Human embryonic kidney 239T (HEK293T)	ATCC	Cat# CRL-3216
Experimental Models: Organisms/Strains		
Rat (Wistar)	Janvier	N/A
Mouse (C57 BL/6JRj)	Janvier	N/A

(*Continued on next page*)

Continued

REAGENT or RESOURCE	SOURCE	IDENTIFIER
Recombinant DNA		
Bio-mCherry	[30]	N/A
Bio-GFP-N1	[31]	N/A
pβactin-GFP	[32]	N/A
pGW1-GFP	[32]	N/A
pSuper vector	[33]	N/A
pβactin-GFP-FRB	[34]	N/A
pGW1-PEX-RFP-FKBP	[34]	N/A
Protein-biotin ligase BirA	[35]	N/A
GFP-MACF18	[10]	N/A
mCherry-α-tubulin	[10]	N/A
GFP-CAMSAP1	[10]	N/A
GFP-CAMSAP2	[10]	N/A
GFP-CAMSAP3	[10]	N/A
GFP-CAMSAP2-CC-CKK	[10]	N/A
GFP-CAMSAP2-N1	This paper	N/A
GFP-CAMSAP2-N2	This paper	N/A
GFP-CAMSAP2-N3	This paper	N/A
GFP-CAMSAP2-MBD-CKK	This paper	N/A
3GFP-CAMSAP2	[10]	N/A
Dox-inducible PA-GFP-α-tubulin	This paper	N/A
Bio-mCherry-KIFC3-CAMSAP2 chimera	This paper	N/A
Bio-mCherry-EMTB-CC-MD chimera	This paper	N/A
EMTB-3GFP	Addgene	Cat# 26741; RRID: Addgene_26741
Bio-mCherry-KIFC3-FL-WT	This paper	N/A
Bio-mCherry-KIFC3-N1	This paper	N/A
Bio-mCherry-KIFC3-N2	This paper	N/A
Bio-mCherry-KIFC3-N3	This paper	N/A
Bio-mCherry-KIFC3-N4	This paper	N/A
Bio-mCherry-KIFC3-N5	This paper	N/A
Bio-mCherry-KIFC3-CC-MD	This paper	N/A
Bio-mCherry-KIFC3-Rigor	This paper	N/A
Bio-mCherry-KIFC3-FL (mouse shRNAs resistant)	This paper	N/A
KIFC3-CC-MD-GFP-FRB	This paper	N/A
KIFC3-CC-MD-Rigor-GFP-FRB	This paper	N/A
Oligonucleotides		
See Table S2.	N/A	N/A
Software and Algorithms		
ImageJ	NIH	https://imagej.nih.gov/ij/; RRID: SCR_003070
NeuronJ	[36]	http://www.imagescience.org/meijering/software/neuronj/; RRID:SCR_002074
GraphPad Prism 8	GraphPad	https://www.graphpad.com/scientific-software/prism/; RRID: SCR_002798
Kymoreslicewide	Github	https://github.com/ekatrukha/KymoResliceWide

LEAD CONTACT AND MATERIALS AVAILABILITY

Plasmids generated in this study are available on request. Further information and requests for resources and reagents should be directed to and will be fulfilled by the Lead Contact, Casper Hoogenraad (c.hoogenraad@uu.nl).

EXPERIMENTAL MODEL AND SUBJECT DETAILS

Animals

All animal experiments were performed in compliance with the guidelines for the welfare of experimental animals issued by the Federal Government of the Netherlands, and were approved by the Animal Ethical Review Committee (DEC) of Utrecht University (permit number 2014.I.03.020 and AVD1080020173404).

Cell line culture and transfection

HEK293T were used for biochemistry experiments and cultured in DMEM/Ham's F10 (50:50) supplemented with 10% FCS and 1% penicillin/streptomycin at 37 °C and 5% CO2. HEK cells were transfected with PEI (Polyethylenimine HCl MAX Linear MW 40000 (PolySciences, 24765–2)). Cells were spitted 24hour before transfection with 1:3 dilutions. The next day, PEI (1 μg/μL): DNA (1 μg/μL) (3:1) were mixed in Ham's F10 and incubated for 5 minutes at room temperature. The mixture was added to cells and incubated overnight. Cells were harvest after 24 hours for the pull-down experiments.

COS7 cells were used for localization studies and cultured in DMEM supplemented with 10% FCS and 1% penicillin/streptomycin at 37 °C and 5% CO2. For immunocytochemistry experiments, COS7 cells were transfected with FuGENE6. Cells were spitted 24hour before transfection with 1:5 dilutions. The next day, Fugene6: DNA (1 μg/μL) (3:1 vol) were mixed in DMEM and incubated for 5 minutes at room temperature. The mixture was added to cells with DMEM empty medium and incubated for 30mins. Then, the empty medium was replaced by the full culture medium. Cells were fixed after 24 hours for staining and imaging. All cell lines were routinely tested negative for mycoplasma.

Primary neuron culture and transfection

Primary hippocampal neurons were prepared from embryonic 18 rat brain, dissociated with Trypsin at 37 degree and plated on coverslips pre-coated with Poly-L-lysine (37.5 μg/ml, Sigma) and Laminin (1.25 μg/ml, Sigma). Neurons were cultured in Neurobasal medium (Invitrogen) containing 2% B27 (Invitrogen), 0.5 mM glutamine (Invitogen), 15.6 μM glutamate (Sigma) and 1% penicillin/streptomycin (Invitrogen) at a density of 100,000/coverslip.

All neurons were transfected with Lipofectamine 2000 (ThermoFisher, 11668019) mixed with proper DNA. For knock-down experiments, neurons were transfected 3 days before fixation with 1.5 μg shRNA constructs and 0.5 μg pβactin-GFP construct as fill or 0.25 μg GFP-MT+TIP to visualize microtubule plus-end tips per well. And for normal overexpression experiments, neurons were transfected 24 hours before fixation with 1 μg in total specified constructs. First, neurons were incubated with the mixture in NB with 15.6 μM glutamate at 37 °C in 5% CO2 for 45 minutes. Washed once with NB and then neurons were transferred to their original medium.

Neuron transfected with CRISPR knock-in construct at DIV4 and fixed at DIV11. GFP was knocked in rat genomic DNA after KIFC3 coding sequence. GFP antibody was used to enhance signal and Trim46 were stained to visualize AIS.

Ex vivo electroporation and organotypic brain slice cultures

Pregnant C57BL/6 mice at E14.5 were sacrificed by cervical dislocation. Embryonic mice brains were electroporated with 1.5μl DNA mixture containing a MARCKS-GFP together with pSuper-scrambled, KIFC3 shRNAs, or KIFC3 shRNA in the presence of KIFC3 as rescue. The DNA mix was dissolved in MQ with 0.05% FastGreen FCF Dye (Sigma) and injected in the lateral ventricles of the embryonic brains by using borosilicate glass micro-pipettes (World Precision Instruments) and a PLI-100A Picoliter Microinjector (Warner Instruments). The electroporation was conducted using platinum plated electrodes (Nepagene) with an ECM 830 Electro Square Porator (Harvard Apparatus) which was set to 3 unipolar pulses of 100ms at 30V with 100ms intervals. Embryonic brains were then isolated and collected in ice-cold cHBSS, embedded in 3% SeaPlaque GTG Agarose (Lonza) in cHBSS and sectioned coronally into 300 μm thick slices using a VT1000 S Vibratome (Leica). Slices were collected on Poly-L-lysine and Laminin-coated culture membrane inserts (Falcon), placed on top of slice culture medium (70% v/v Basal Eagle Medium, 26% v/v cHBSS, 20mM D-glucose, 1mM L-glutamine, 0.1 mg/mL penicillin/streptomycin) and cultured 4 days prior to fixation.

METHOD DETAILS

DNA and shRNA constructs

CAMSAP2 shRNA has been previously described [10]. All KIFC3 shRNAs target sequences and CRISPR KI target sequences were shown in Table S2.

Bio-mCherry [30] and Bio-GFP-N1 [31] vectors are gifts from Dr. Anna Akhmanova (Utrecht University). The following plasmids have been described: pβactin-GFP, pGW1-GFP [32], pSuper vector [33], pβactin-GFP-FRB, pGW1-PEX-RFP-FKBP [34], protein-biotin ligase BirA [35], GFP-MT+TIP, mCherry-α-tubulin, GFP-CAMSAP1, GFP-CAMSAP2, GFP-CAMSAP3, GFP-CAMSAP2-CC-CKK and 3GFP-CAMSAP2 has been described [10].

For the dox-inducible PA-GFP-α-tubulin construct, Transactivator (rtTA2-M2) is taken from pSIN-TRE-S [37] and it is inserted after CMV promoter. α-Tubulin with photoactivatable GFP tag is sub-cloned from PA-GFP-α-tubulin construct described before [38].

CAMSAP2 truncations and KIFC3-CAMSAP2 chimera construct was cloned by a PCR-base strategy into pGW1-GFP vector. All KIFC3 constructs were generated from Mouse KIFC3 (NM_001145832) (IMAGE:6810893) and cloned by a PCR-base strategy into a Bio-mCherry vector or Bio-GFP-N1 vector.

EMTB-CC-MD chimera construct was cloned by a PCR-base strategy into Bio-mCherry vector. EMTB domain was taken from EMTB-3GFP (Addgene plasmid #26741) [39]. The motor domain was taken from Mouse KIFC3 full length (GenBank: NM_001145832) (IMAGE:6810893).

Coiled coil regions were identified in the protein structure of kinesins using COILS prediction software (https://www.ch.embnet.org/software/COILS_form.html). Gibson assembly system was applied to produce KIFC3-rigor (396T > N) construct. KIFC3-CC-MD-WT and KIFC3-CC-MD-rigor was cloned by PCR into pβactin-GFP-FRB vector.

PCR-base strategy was used and the KIFC3 fragment carrying mutations was cloned into Bio-mCherry vector. The mutations for cloning mouse shRNA resistant KIFC3 didn't lead to amino acid change. The primers were shown in Table S2.

Antibody and reagents

The following antibodies were used for the immunofluorescence staining: mouse anti-α-tubulin (1:800, Sigma-Aldrich, T-5168), mouse anti-acetylated-tubulin (1:400, Sigma-Aldrich, T7451), rat anti-tyrosinated-tubulin (1:400, Abcam, ab6160), chicken anti-MAP2 (1:1,000, Abcam, ab5392), chicken anti-GFP (1:1000, Aves Labs, GFP-1020), rabbit anti-CAMSAP2 (1:200, Proteintech, 17880-1-AP), rabbit anti-KIFC3 (1:400 Santa Cruz, sc-134681), rabbit anti-TRIM46 [29]; mouse anti-MAP2 (1:600, Sigma-Aldrich, M9942) and rabbit anti-mCherry/RFP (1:1,000, Rockland, 600-401-379).

Secondary antibodies were used at 1:1,000 concentrations as follows: Alexa Fluor 405-, Alexa Fluor 488-, Alexa Fluor 568-, Alexa Fluor 647-conjugated secondary antibodies (Life Technologies).

The following antibodies were used for western blot: mouse anti-mCherry (1:1,000 Clonetech, 632543); rabbit anti-GFP (1:2,500 Abcam, ab290); IRDye 680LT-(1:20,000) and IRDye 800CW-conjugated (1:15,000) secondary antibodies (LI-COR Biosciences).

Other reagents used in this study include: Doxycyclin (0.5μg/ml, Sigma-Aldrich), Lipofectamine2000 (Thermofiser, 11668019), PEI (PolySciences, 24765–2), FuGENE 6 (Roche, Cat#11836145001), Vectashield mounting medium (Vectorlabs, H-1000) and Mowiol mounting medium (10% Mowiol 4-88, 25% Glycerol, 0.1M Tris-Cl (0.2M, pH8.5) and 2.5% DABCO).

Biotin-streptavidin pull-down and western blot

Streptavidin pull-down assays were performed by using Dynabeads M-280 streptavidin beads (Invitrogen). For normal pull down, HEK293 cells were transfected with overexpression constructs indicated in figure legend together with BirA. After 24 hours expression, cells were harvested in ice-cold PBS and lysed with lysis buffer (150 mM Tris-HCl pH 7.5, 150 mM NaCl, 1% Triton X-100 and 1x protease inhibitor cocktail). 80% of the total cell lysates were centrifuged at 13,000 rpm for 5 minutes and the supernatants were transferred and incubated with streptavidin beads which were already blocked by 0.2% Chicken Egg Albumine (Sigma). The left cell lysates were denatured with SDS/DTT sample buffer and used as input. After 40 minutes incubation at 4 degree, beads was washed 5 times with normal washing buffer (100mM Tris pH7.5, 150 mM NaCl, 0.5% Triton X-100 and 0.5x protease inhibitor cocktail). Samples were eluted with SDS/DTT sample buffer and boiled for 5 minutes at 95 degree.

For the western blot assays, samples were loaded into 10% SDS-PAGE gels and transferred to nitrocellulose membrane. Membranes were blocked with 2% BSA (bovine serum albumin) in PBS/0.05% Tween 20. Primary antibodies were diluted in blocking buffer and incubated with the membranes overnight at 4°C, washed 3 times with PBS/0.05% Tween 20 and incubated with secondary IRDye 680LT or IRDye 800LT antibodies for 45 minutes at room temperature. Membranes were then washed 3 times with PBS/0.05% Tween 20 and scanned on Odyssey Infrared Imaging system (LI-COR Biosciences).

Biotin-streptavidin pull-down and Mass spectrometry

The first part of the Biotin-streptavidin pull-down for mass spectrometry is the same as the pull-down for protein interaction validation. But instead of 5 times normal wash buffer, 2 times high salt washing buffer (20 mM Tris pH 8.0, 500 mM KCl, 0.1% Triton X-100), 2 times low salt washing buffer (20 mM Tris pH 8.0, 100 mM KCl and 0.1% Triton X-100) and 2 times high salt washing buffer again were used to wash the bead. Brains were obtained from female adult rats and homogenized in tissue lysis buffer (50 mM Tris HCl, 150 mM NaCl, 0.1% SDS, 0.2% NP-40, and protease inhibitors). Brain lysates were centrifuged at 16,000 × g for 15 minutes at 4°C, and the supernatant was then incubated for 1 hour at 4°C with beads previously conjugated with the protein of interest. After second incubation of 1 hour at 4 degree, beads were washed 5 times with normal washing buffer.

For MS analysis, the beads were resuspended in 15 μL of Laemmli Sample buffer (Biorad), boiled at 99°C for 10 minutes and supernatants were loaded on 4%–12% Criterion XT Bis-Tris precast gel (Biorad). The gel was fixed with 40% methanol and 10% acetic acid and then stained for 1h using colloidal Coomassie dye G-250 (Gel Code Blue Stain Reagent, Thermo Scientific). Each lane from the gel was cut in 3 slices, destained and digested using trypsin. In brief, each lane from the gel was cut into three pieces and placed in 0.5ml tubes. Gel pieces were then washed with 250 μL of water, followed by 15 minutes dehydration in acetonitrile. Proteins were reduced (10 mM dithiothreitol, 1h at 56°C), dehydrated and alkylated (55 mM iodoacetamide, 1h in the dark). After two rounds of dehydration, trypsin (Promega) was added to the samples (20 μL of 0.1 mg/ml trypsin in 50 mM Ammoniumbicarbonate) and incubated overnight at 37°C. Peptides were extracted with ACN, dried down and reconstituted in 10% formic acid prior MS analysis [40, 41].

Immunofluorescence fixation and staining

Extraction, fixation and immunocytochemistry were performed as previously described [42]. For COS7 without staining, cells were fixed in 4% paraformaldehyde, washed 3 times in PBS. For COS7, which need to be stained, cells were further permeabilized with 0.25% Triton X-100, blocked with 2% w/v bovine serum albumin (BSA) in PBS and incubated with primary antibodies in PBS with 2% BSA overnight at 4 degree.

For neurons, cells were fixed with 4% PFA (paraformaldehyde) for 10-15 minutes, washed 3 times with PBS (phosphate buffer saline), and incubated with primary antibodies in GDB buffer (0.2% BSA, 0.8 M NaCl, 0.5% Triton X-100, 30 mM phosphate buffer) overnight at 4 degree. For super-resolution imaging, cells were incubated for 90 s in an extraction buffer preheated at 37°C (80 mM pipes, 2 mM MgCl2, 1 mM EGTA, 0.3% Triton X-100 and 0.25% glutaraldehyde, pH 6.9), followed by incubation with 4% PFA preheated at 37°C for 10 minutes. Neurons were further permeabilized with 0.25% Triton X-100 and blocking was performed with 2% w/v bovine serum albumin (BSA), in PBS, pH 7.4. Primary antibodies were incubated overnight at 4 degree in blocking buffer.

For neurons and COS7, secondary antibodies were incubated 1 hour at room temperature. Mowiol or Vectashield mounting medium was used for mounting.

Slice immunofluorescence and imaging

Organotypic slices were fixed with 4% paraformaldehyde in PBS. Fixed slices were then permeabilized and blocked in 10% normal goat serum/0.2% Triton X-100/PBS. GFP signal was amplified by incubating with anti-GFP antibody overnight, followed by secondary Alexa 488 antibody and DAPI incubation overnight. Slices were washed 4 times for 15 min in PBS. Slices were mounted using Vectashield mounting medium (Vector Laboratories) with DAPI. Z stack acquisitions were taken along dorsal telencephalon covering the transfected area using a LSM700 (Zeiss) confocal microscope equipped with a Plan-Apochromat 20x NA 0.8 objective with a 0.5x magnification.

Laser Scanning Confocal and STED microscopy

After immunofluorescence staining, neurons and COS7 cells were imaged using Zeiss LSM700 confocal laser scanning microscope with 63x NA 1.4 oil objective. Gated STED imaging was performed with Leica TCS SP8 STED 3 × microscope using HC PL APO × 100/1.4 oil STED WHITE objective.

Live cell imaging

All the live imaging was performed on a Nikon Eclipse TE2000E inverted microscope equipped with Evolve 512 EMCCD camera (Roper Scientific), spinning disk confocal (Roper Scientific), incubate chamber (Tokai Hit) and MetaMorph 7.7.5 software (Molecular Device) was used for all the live imaging experiments.

GFP-MT+TIP tracking

GFP-MT+TIP construct was co-transfected with proper shRNA in DIV9 neurons and imaged at DIV12. Around 20 μm region was selected from middle proximal dendrites and imaged with 1frame/second for 5 minutes. ImageJ plugin KymographWideSlice was used to analyze microtubule comets movement.

Photo-ablation experiments in neurons

A Teem Photonics 355 nm Q-switched pulsed laser was used for photo-ablation on the described spinning disk microscope. For microtubule orientation experiments, microtubules were photo-ablated in proximal dendrites and the 10 μm region before and after was imaged with 1 frame/second for 5 minutes.

Microtubule photo ablation in COS7

COS7 cells were transfected with 3GFP-CAMSAP2 and mCherry-KIFC3. 100nM of SIR-tubulin (Spirochrome) was added to the medium and incubated overnight to visualize microtubules. Cells were imaged using Eclipse 80i(Nikon) microscope equipped with an Apo 100x 1.49 N.A. Oil objective Photometrics Evolve 512 EMCCD camera (Roper scientific) and perfect focus system and iLas2 laser illumination system (Roper Scientific France).

The 532nm Q-switched pulsed laser (Teem Photonics) was used for photoablation. Microtubules were severed using 532 nm laser and then imaged with 7 s intervals for total 5 min to track CAMSAP2 and KIFC3 localization at microtubule minus end.

Photoactivation experiments

The Dox-inducible PA-GFP-α-Tubulin with proper shRNAs was co-transfected in DIV9 neurons and Doxycycline was added the next day. Neurons were imaged at DIV12. Photoactivation of PA-GFP-α-Tubulin was performed on the FRAP setup and 405 nm laser was used for activation. Around 15 μm regions were selected from middle dendrite and it was imaged every 15minutes for 3 hours. The elongation of the selected region during imaging is regarded as microtubule sliding.

QUANTIFICATION AND STATISTICAL ANALYSIS

All statistical details including the definitions of n, numbers of n and statistical tests performed can be found in each Figures and Figure legends. GraphPad were used for graphs and statistics. t test (column graph) or 2-way Anova (line graph) test was performed for statistics and $p < 0.05$ was considered significant (*$p < 0.05$; **$p < 0.01$; *** $p < 0.001$.). Image processing and analysis were performed by using ImageJ. Secondary structure of KIFC3 was defined by using SMART (Simple Modular Architecture Research Tool; at EMBL) and COILS (Prediction of coiled coil regions in proteins, Lupas's method, at EMBnet-CH).

Sholl analysis

ImageJ plug-in, Sholl analysis and NeuronJ [36], was used for neuron morphology analysis. The distance is measured from outer edge of soma. The interval is 5 μm and 4 data points were averaged for line graph.

Quantification of *ex vivo* neuronal migration and morphology

The relative positions of the cell bodies of GFP-positive neurons along the radial axis starting from 30% above ventricular to pial surface were measured by ImageJ plugin (Analyze Particle). The relative position of neurons in the regions of interest was recorded in terms of distance between the ventricular and the pial surface. The positional information of all transfected neurons, together with the top (pial surface) and bottom (ventricular surface) boundaries were exported to Excel. Based on the relative position of each neuron and the total numbers of neurons counted, the radial cell distribution along the radial axis was obtained by further data processing using an Excel macro and presented as percentage of migration.

Regions representing 20% of the cortical width below the pial were cropped for morphology analysis. Neurons were classified into 4 types: type 1 with no leading process, type 2 with 1 leading process, type 3 with secondary branches, type 4 with branches over 2. Counting was performed using ImageJ plugin (Analyze Particle).

Mass spectrometry analysis

All samples were analyzed with an Agilent 1290 Infinity LC (Agilent Technologies), operating in reverse-phase (C18) mode, coupled to a TripleTOF 5600 (Ab Sciex). Peptides were loaded onto a trap column (Reprosil pur C18, Dr. Maisch, 100 μm x 2 cm, 3 μm; constructed in-house) with solvent A (0.1% formic acid in water) at a maximum pressure of 800 bar and chromatographically separated over the analytical column (Poroshell 120 EC C18, Agilent Technologies, 50 μm x 50 cm, 2.7 μm) using 90 minutes linear gradient from 7%–30% solvent B (0.1% formic acid in acetonitrile) at a flow rate of 150 nL/min. MS spectra (350-1250 m/z) were acquired in high-resolution mode (R > 30,000), whereas MS2 was in high-sensitivity mode (R > 15,000). For data analysis, raw files were processed using Proteome Discoverer 1.4 (version 1.4.1.14, Thermo Scientific). Database search was performed using the Uniprot rat database and Mascot (version 2.5.1, Matrix Science, UK) as the search engine. Carbamidomethylation of cysteines was set as a fixed modification and oxidation of methionine was set as a variable modification. Trypsin was set as cleavage specificity, allowing a maximum of 2 missed cleavages. Data filtering was performed using a percolator, resulting in 1% false discovery rate (FDR). Additional filters were search engine rank 1 and mascot ion score > 20.

Analysis of polarity index

KIFC3 and truncations were expressed in DIV10 neurons, fixed at DIV11, stained for TRIM46 as axon marker. Average intensity of 15 μm in the proximal axon or dendrite was measured. At least 2 dendrites were included, and background was subtracted. Polarity index was calculated with the formula: PI = (Ia-Id)/(Id+Ia). Id corresponds to mean dendrite intensity, while Ia is the mean proximal axon intensity. PI > 0 indicates the polarization is biased toward axon and PI < 0 to the dendrite.

Microtubule ablation in COS7

Images were processed using FIJI software. Background intensity was subtracted from each frame of the time-lapse movies. Gaussian Blur filter of 2 pixels was applied. After microtubule severing, ROI of 10x10 pixels was selected at the end of the microtubule minus end to measure the intensity of CAMSAP2 and KIFC3 in each frame. Intensity was normalized to the maximum value of the time-lapse acquisition. For plotting (Figure 2M) moving time average of 5 frames was calculated.

PEX quantification

At least, six cells were analyzed per condition. Images were processed for background subtraction. Cell contour was manually traced using the motor channel and cell center was determined. Peroxisome distribution was measured by radial intensity analysis. The radius of the circle containing up to 90% peroxisome intensity was determined and was normalized by radius of the circle containing the entire cell. The normalized radius was used as the parameter for peroxisome distribution in Figure S3C.

DATA AND CODE AVAILABILITY

The published article includes all datasets generated or analyzed during this study. Additional requests should be sent to the Lead Contact, Casper Hoogenraad (c.hoogenraad@uu.nl).

Current Biology, Volume *30*

Supplemental Information

Microtubule Minus-End Binding Protein CAMSAP2 and Kinesin-14 Motor KIFC3 Control Dendritic Microtubule Organization

Yujie Cao, Joanna Lipka, Riccardo Stucchi, Mithila Burute, Xingxiu Pan, Sybren Portegies, Roderick Tas, Jelmer Willems, Lena Will, Harold MacGillavry, Maarten Altelaar, Lukas C. Kapitein, Martin Harterink, and Casper C. Hoogenraad

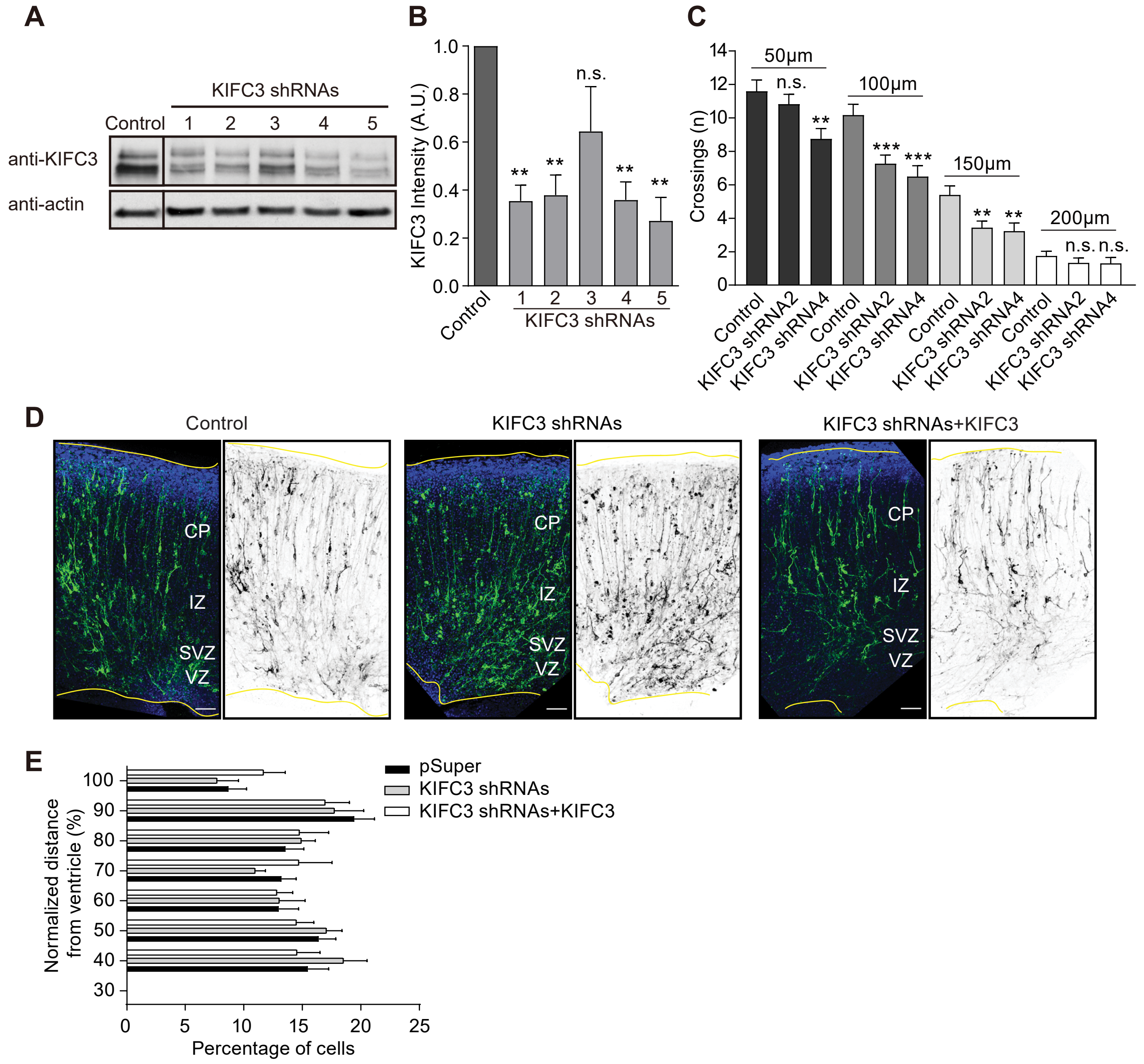

Figure S1. **KIFC3 is important for dendrite branching. Related to Figure 1.**

(A) Example of a western blot of protein extracts of DIV 7 hippocampal neurons electroporated at DIV 0 with pSuper-scrambled as Control or KIFC3-shRNA1, 2, 3, 4 and 5. Levels of actin were used as loading Control.

(B) Quantification of protein levels of KIFC3 of samples represented in (A) (N=3). Error bars represent SEM. *p < 0.05; **p < 0.01; *** p < 0.001 (Unpared T-test).

(C) Quantification of crossings from the sholl analysis in Figure 1B at 50µm, 100µm, 150µm and 200µm positions. Error bars represent SEM. Unpared T-test was performed and columns were compared with corresponding Control. *p < 0.05; **p < 0.01; *** p < 0.001.

(D-E) Representative images of mouse cortex after 4 days of *ex vivo* electroporated with GFP and pSuper-scrambled, KIFC3 shRNAs or KIFC3 shRNAs with KIFC3 as rescue. Left panels represent GFP and DAPI channels. DAPI is in blue and GFP is in Green. Pail surface at the top and ventricle at the bottom are outlined in yellow. CP, cortical plate; IZ, intermediate zone;SVZ, subventricular zone; VZ, ventricular zone. Right panels represent GFP only channel and GFP is in gray. Scale bars=50µm. Error bars represent SEM.

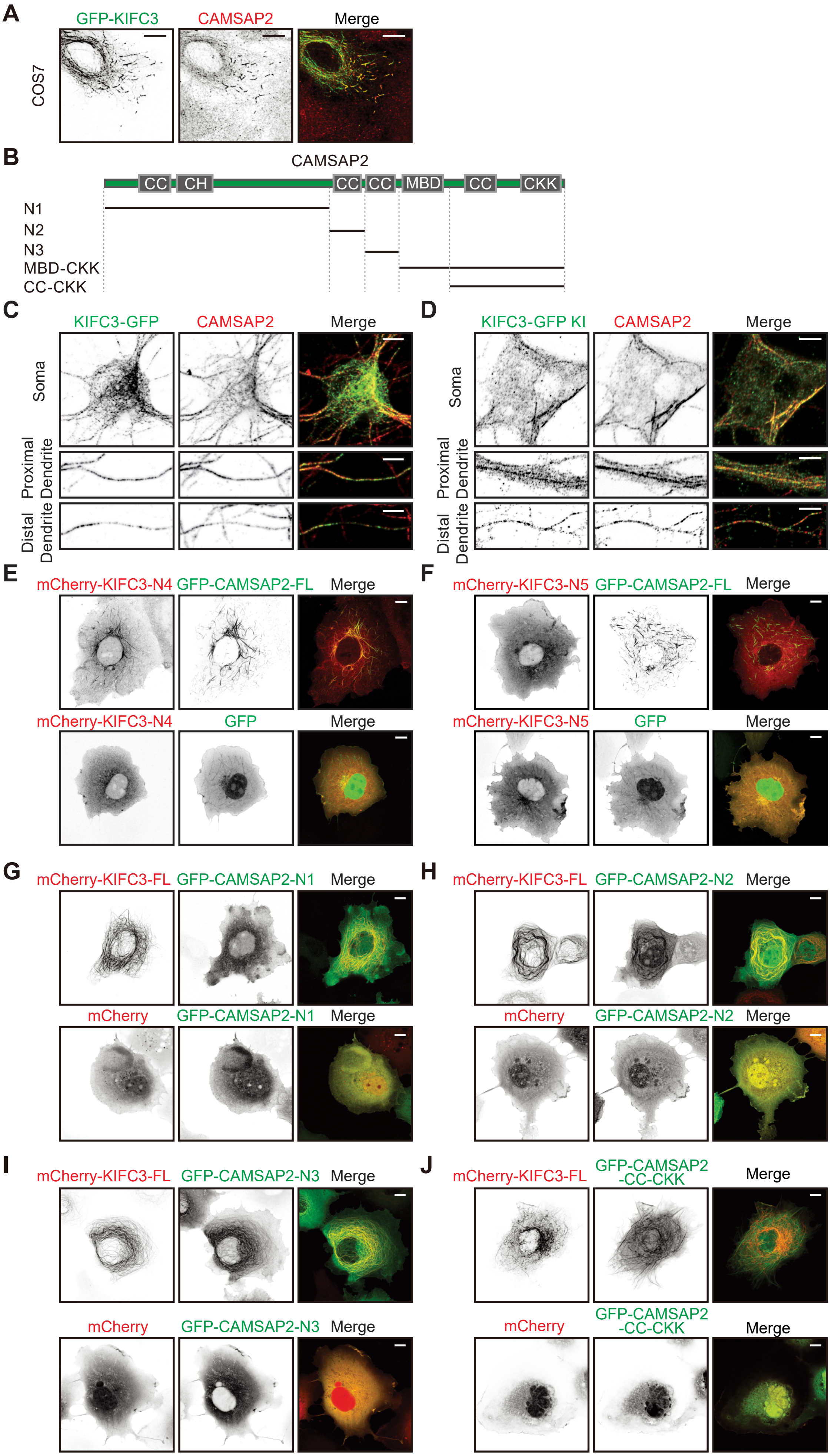
A
GFP-KIFC3
CAMSAP2
Merge
COS7
B
CAMSAP2
CC
CH
CC
CC
MBD
CC
CKK
N1
N2
N3
MBD-CKK
CC-CKK
C
KIFC3-GFP
CAMSAP2
Merge
Soma
Proximal Dendrite
Distal Dendrite
D
KIFC3-GFP KI
CAMSAP2
Merge
Soma
Proximal Dendrite
Distal Dendrite
E
mCherry-KIFC3-N4
GFP-CAMSAP2-FL
Merge
mCherry-KIFC3-N4
GFP
Merge
F
mCherry-KIFC3-N5
GFP-CAMSAP2-FL
Merge
mCherry-KIFC3-N5
GFP
Merge
G
mCherry-KIFC3-FL
GFP-CAMSAP2-N1
Merge
mCherry
GFP-CAMSAP2-N1
Merge
H
mCherry-KIFC3-FL
GFP-CAMSAP2-N2
Merge
mCherry
GFP-CAMSAP2-N2
Merge
I
mCherry-KIFC3-FL
GFP-CAMSAP2-N3
Merge
mCherry
GFP-CAMSAP2-N3
Merge
J
mCherry-KIFC3-FL
GFP-CAMSAP2 -CC-CKK
Merge
mCherry
GFP-CAMSAP2 -CC-CKK
Merge

Figure S2. KIFC3 interacts with CAMSAP2. Related to Figure 2.

(A) Representative images of a COS7 cell transfected with KIFC3-GFP and stained with CAMSAP2 antibody. Scale bar=10μm.

(B) Schematic representation of CAMSAP2 truncation constructs used. CC: coiled-coiled domain, MD: motor domain, CH: Calponin-homology domain, MBD: microtubule binding domain, CKK: CKK domain.

(C) Representative images of a DIV7 neuron transfected with KIFC3-GFP and stained with CAMSAP2 antibody. Scale bar=5μm.

(D) Representative images of a DIV11 neuron transfected with the HITI CRISPR GFP knock-in construct and probed for CAMSAP2 and GFP (to enhance the signal in transfected cells). Scale bar=5μm.

(E-F) Representative images of COS7 cells transfected with GFP-CAMSAP2-FL or GFP and co-transfected with mCherry-KIFC3-N4 or mCherry-KIFC3-N5. Scale bar=10μm.

(G-J) Representative images of COS7 cells transfected with either mCherry or mCherry-KIFC3 together with GFP tagged CAMSAP2 truncations. Scale bar=10μm.

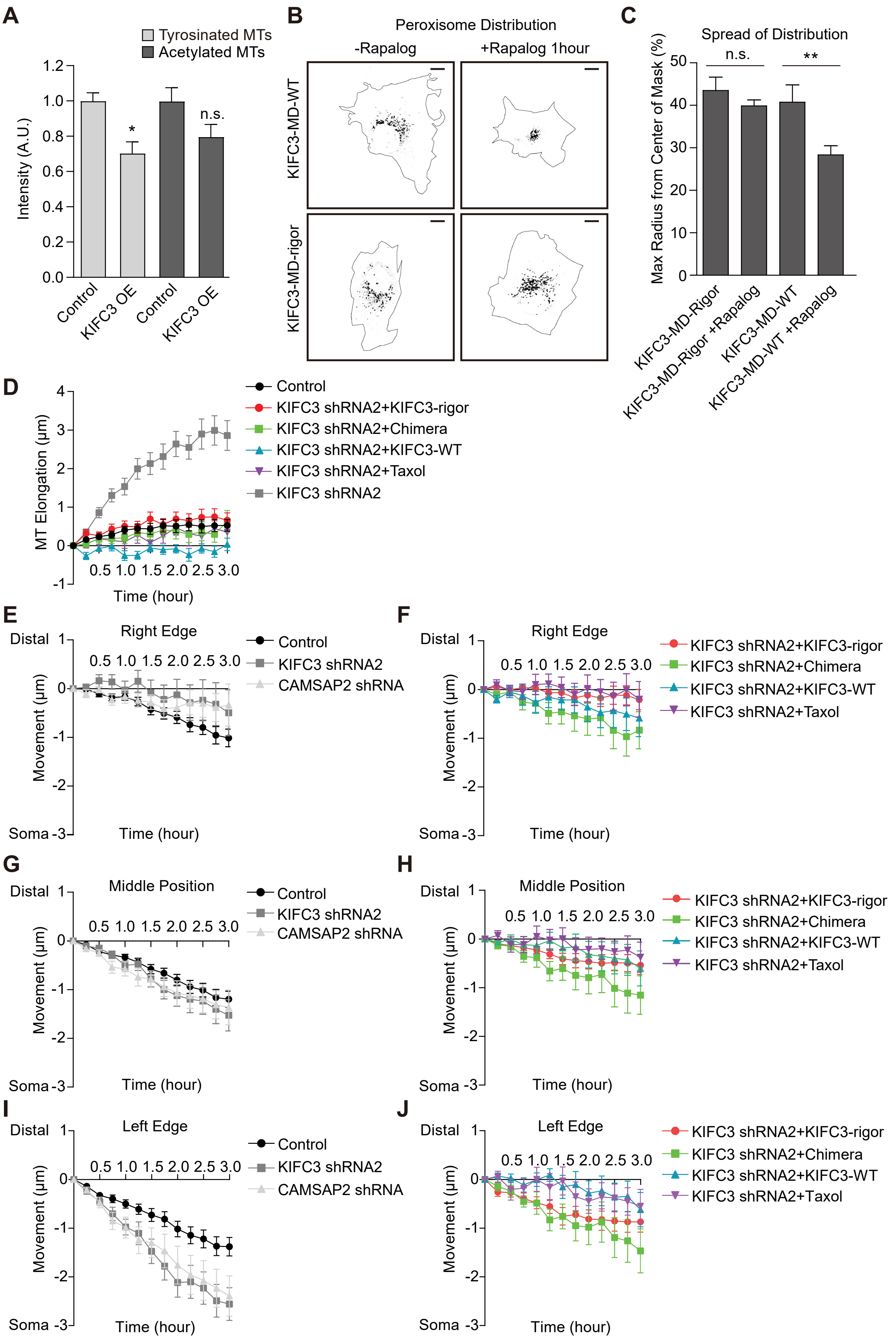

A
Tyrosinated MTs
Acetylated MTs
Intensity (A.U.)
1.2
1.0
0.8
0.6
0.4
0.2
0.0
*
n.s.
Control
KIFC3 OE
Control
KIFC3 OE
B
Peroxisome Distribution
-Rapalog
+Rapalog 1hour
KIFC3-MD-WT
KIFC3-MD-rigor
C
Spread of Distribution
Max Radius from Center of Mask (%)
50
40
30
20
10
0
n.s.
**
KIFC3-MD-Rigor
KIFC3-MD-Rigor +Rapalog
KIFC3-MD-WT
KIFC3-MD-WT +Rapalog
D
MT Elongation (μm)
4
3
2
1
0
-1
0.5 1.0 1.5 2.0 2.5 3.0
Time (hour)
Control
KIFC3 shRNA2+KIFC3-rigor
KIFC3 shRNA2+Chimera
KIFC3 shRNA2+KIFC3-WT
KIFC3 shRNA2+Taxol
KIFC3 shRNA2
E
Right Edge
Distal 1
0
-1
-2
Soma -3
Movement (μm)
0.5 1.0 1.5 2.0 2.5 3.0
Time (hour)
Control
KIFC3 shRNA2
CAMSAP2 shRNA
F
Right Edge
Distal 1
0
-1
-2
Soma -3
Movement (μm)
0.5 1.0 1.5 2.0 2.5 3.0
Time (hour)
KIFC3 shRNA2+KIFC3-rigor
KIFC3 shRNA2+Chimera
KIFC3 shRNA2+KIFC3-WT
KIFC3 shRNA2+Taxol
G
Middle Position
Distal 1
0
-1
-2
Soma -3
Movement (μm)
0.5 1.0 1.5 2.0 2.5 3.0
Time (hour)
Control
KIFC3 shRNA2
CAMSAP2 shRNA
H
Middle Position
Distal 1
0
-1
-2
Soma -3
Movement (μm)
0.5 1.0 1.5 2.0 2.5 3.0
Time (hour)
KIFC3 shRNA2+KIFC3-rigor
KIFC3 shRNA2+Chimera
KIFC3 shRNA2+KIFC3-WT
KIFC3 shRNA2+Taxol
I
Left Edge
Distal 1
0
-1
-2
Soma -3
Movement (μm)
0.5 1.0 1.5 2.0 2.5 3.0
Time (hour)
Control
KIFC3 shRNA2
CAMSAP2 shRNA
J
Left Edge
Distal 1
0
-1
-2
Soma -3
Movement (μm)
0.5 1.0 1.5 2.0 2.5 3.0
Time (hour)
KIFC3 shRNA2+KIFC3-rigor
KIFC3 shRNA2+Chimera
KIFC3 shRNA2+KIFC3-WT
KIFC3 shRNA2+Taxol

Figure S3. KIFC3-N1 shows dominant negative effect, KIFC3-rigor mutation validation and microtubule mobility rescue and quantification. Related to Figure 3 and Figure 4.

(A) Quantification of acetylated tubulin, tyrosinated tubulin levels in DIV11 neurons transfected with mCherry-KIFC3. Neighboring non-transfected neurons were used as Control. N=2. Control: n=22; mCherry-KIFC3: n=20. Error bars represent SEM. Unpared-T-test was performed and columns were compared with corresponding Control. $*p < 0.05$; $**p < 0.01$; $*** p < 0.001$.

(B) Representative images of COS7 cells transfected with either PEX-RFP-FKBP and KIFC3-CC-MD-GFP-FRB or PEX-RFP-FKBP and KIFC3-CC-MD Rigor (396T-N)-GFP-FRB. PEX-RFP-FKBP was used to visualize peroxisomes in cells. Scale bar=10 μm.

(C) Quantification of peroxisome redistribution in cells described in (B). KIFC3-MD-Rigor: N=1, n=7; KIFC3-MD-Rigor+Rapalog: N=1, n=6, KIFC3-MD-WT: N=1, n=6; KIFC3-MD-WT+Rapalog: N=1, n=12. Error bars represent SEM. $*p < 0.05$; $**p < 0.01$; $*** p < 0.001$ (Unpaired T-test).

(D) Quantification of microtubule elongation corresponding to Figure 4A, 4C and 4D. Control: N=5, n=36; KIFC3 shRNA2: N=4, n=35; KIFC3 shRNA2+KIFC3-rigor: N=2, n=20; KIFC3 shRNA2+KIFC3-Chimera: N=3, n=10; KIFC3 shRNA2+KIFC3-WT: N=3, n=12; KIFC3 shRNA2+Taxol: N=3, n=8. Error bars represent SEM.

(E-J) Quantification of microtubule movement of the left edge, right edge and middle position of the photo-converted region corresponding to Figure 4A, 4C and 4D. Error bars represent SEM.

		Control		KIFC3-FL		KIFC3-N1		KIFC3-N3		
**PROTID**	**Name**	**Peptides**	**PSM**	**Peptides**	**PSM**	**Peptides**	**PSM**	**Peptides**	**PSM**	
Q4KLH6	CEP162	0	0	9	17	10	21	9	17	MT/Centrosome
Q63560	Map6	0	0	9	12	0	0	0	0	MT
D4AEC2	Camsap2	0	0	7	7	4	4	0	0	MT
Q63170	Dnah7	0	0	17	27	14	20	0	0	Dynein-ralated
E9PSL8	Kif6	0	0	6	37	1	32	5	48	Kinesin
G3V6L4	Kif5c	1	1	4	5	4	4	4	5	Kinesin
D3Z9C7	Piccolo	0	0	14	18	24	30	12	21	presynapse
F1M8A4	Liprinα	0	0	14	20	7	10	5	7	presynapse
Q9JIR4	Rims1	0	0	10	11	11	12	6	7	presynapse
F1M3B4	UNC13b/MUNC13	0	0	7	8	8	8	0	0	presynapse
R9PY00	Vamp2	0	0	5	5	4	4	0	0	presynapse
P21707	Synaptotagmin1	0	0	4	8	4	6	0	0	presynapse
B5DEH2	Erlin2	1	1	8	9	2	3	0	0	ER
D4ABS2	Dnml1	0	0	9	10	11	14	2	3	Membrane-related
O08679	Mark2	0	0	19	23	0	0	0	0	kinase
P63086	Mapk1	0	0	8	9	2	2	0	0	kinase
B5DFK6	Ap3d1	0	0	10	16	5	7	0	0	AP-3 complex
P62944	Ap2b1	0	0	15	18	6	9	3	3	AP-2 complex

Table S1. Mass Spectrometry Analysis of KIFC3 Interacting Proteins. Related to Figure 2.

Data from affinity purification mass spectrometry analyses (AP-MS) of KIFC3 and truncations in rat brain extracts. PROTID: Uniprot accession code; Name: corresponding gene name; Peptides: peptide matches; PSM: peptide spectrum matches.

Oligonucleotides	Source	Identifier
**KIFC3 shRNAs and CRISPR KI target sequences**		
pSuper-rat KIFC3 shRNA#1 targeting sequence: cgagaacccaggcattaat	This paper	N/A
pSuper-rat KIFC3 shRNA#2 targeting sequence: cagctccgggacaggttat	This paper	N/A
pSuper-rat KIFC3 shRNA#3 targeting sequence: ccacctgttaagtatgtca	This paper	N/A
pSuper-rat KIFC3 shRNA#4 targeting sequence: ctccctaaatcctatttaa	This paper	N/A
pSuper-rat KIFC3 shRNA#5 targeting sequence: cgcatcagctgacaataca	This paper	N/A
pSuper-mouse KIFC3 shRNA#1 targeting sequence: cagctccgtgacaagctgt	This paper	N/A
pSuper-mouse KIFC3 shRNA#2 targeting sequence: ggcttcaatgtctgtatct	This paper	N/A
pSuper-mouse KIFC3 shRNA#3 targeting sequence: ttccaaactcacctacctg	This paper	N/A
CRISPR KI rat KIFC3 targeting sequence: cggctgcagtttccttcgga	This paper	N/A
**Primers for mouse shRNA resistant KIFC3**		
P1-fw: tcgcggccgcctcgagctcaagcttatggtggaga	This paper	N/A
P1-rev: acagcttgtccctcaattggctgctctcctggctg	This paper	N/A
P2-fw: ccaattgagggacaagctgtcccagctgc	This paper	N/A
P2-rev: ggaacacgtcctgttggcttgcccacggggagaagacc	This paper	N/A
P3-fw: aagccaacaggacgtgttccaggaggtgcaggccctcattacctcctgcatcgatggatttaac	This paper	N/A
P3-rev: gttaaatccatcgatgcaggaggtaatgagggcctgcacctcctggaacacgtcctgttggctt	This paper	N/A
P4-fw: cctgcatcgatggatttaacgtctgtatctttgcttacggc	This paper	N/A
P4-rev: tcagcttgctattgcggaagggcacatg	This paper	N/A
P5-fw: cttccgcaatagcaagctgacctacctgctgcaggac	This paper	N/A
P5-rev: atcccgggcccgcggtaccgtcgactcaggctgacggctgcag	This paper	N/A

Table S2. Oligonucleotides. Related to Oligonucleotides section of the Key Resources Table of the STAR Methods.
